# Early Detection and Inhibition of Post‐Surgical Cancer Recurrence by Synthetic Extracellular Vesicles

**DOI:** 10.1002/advs.202523388

**Published:** 2026-04-09

**Authors:** Junli Zhang, Wenjia Chang, Ruolin Hu, Peiyan Su, Qin Zhou, Yanan Li, Zhenzhong Zhang, Kaixiang Zhang

**Affiliations:** ^1^ School of Pharmaceutical Sciences Henan Key Laboratory of Nanomedicine for Targeting Diagnosis and Treatment Zhengzhou University Zhengzhou China; ^2^ Institute of Biomedical Engineering College of Life Sciences Qingdao University Qingdao China; ^3^ State Key Laboratory of Metabolic Dysregulation & Prevention and Treatment of Esophageal Cancer Tianjian Laboratory of Advanced Biomedical Sciences Zhengzhou University Zhengzhou China; ^4^ Key Laboratory of Advanced Drug Preparation Technologies Ministry of Education of China Zhengzhou China

**Keywords:** cancer recurrence, early detection, extracellular vesicles, synthetic biomarkers

## Abstract

Early detection of post‐surgical cancer recurrence could improve patient survival. Endogenous biomarkers remain at the forefront of early detection efforts, but many lack the requisite sensitivity and specificity to effectively guide clinical management. Herein, we employed a synthetic biomarker approach by embedding a tumor specific promoter‐driven synthetic extracellular vesicles (EVs)‐generating system at the tumor resection site. This system reprograms tumor cells to secrete synthetic EVs expressing engineered miR‐26a (E‐miR‐26a, a synthetic barcoding sequence) or PD‐1 (a PD‐L1‐blocking agent), thereby achieving early detection and inhibition of post‐surgical tumor recurrence. In a mouse model of post‐resection tumor recurrence, we demonstrated that monitoring of E‐miR‐26a‐expressing EVs in blood facilitated more timely detection of recurrence than bioluminescence imaging. We further validated that the strategy could detect tumors at early stage across mice with varying tumor burdens. Furthermore, PD‐1‐expressing EVs could bind to PD‐L1 on tumor cells, thereby enhancing T cells activation and antitumor efficacy. Collectively, our findings provide an integrated strategy for the early detection and treatment of post‐surgical tumor recurrence, with the potential to improve long‐term outcomes for cancer patients.

## Graphical abstract

An implantable hydrogel is designed to hold gene transfection agents engineered to turn early recurrent tumor cells into generators of synthetic EVs. These synthetic EVs can express engineered miR‐26a (E‐miR‐26a) for highly sensitive detection and PD‐1 (a PD‐L1‐blocking agent) for therapeutic intervention, thereby enabling early detection and inhibition of post‐surgical tumor recurrence.

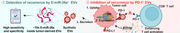

## 1 Introduction

Surgical resection remains the primary and most effective treatment for most solid tumors [1]. However, despite advances in surgical technique and instruments, 30%–40% of patients experience recurrence within five years [2, 3]. Early detection of postoperative recurrence is critical for improving patient outcomes in the adjuvant setting [4, 5, 6]. Current post‐resection surveillance primarily relies on imaging techniques such as computed tomography (CT) and magnetic resonance imaging (MRI) [7, 8, 9]. Yet, conventional imaging lacks the specificity to distinguish tumor recurrence from postoperative changes like inflammation or radiation‐induced necrosis [10, 11, 12]. To address this challenge, our group has previously explored implantable hydrogel sensors for localized recurrence monitoring, [13, 14] including a PD‐L1 aptamer‐functionalized DNA hydrogel that captures relapsed tumor cells in situ while amplifying ATP signals for early detection [14]. However, due to the limited penetration depth of in situ fluorescence signals in vivo, there remains an urgent clinical need for reliable tumor biomarkers with high signal‐to‐noise ratios to improve early recurrence detection.

Recently, emerging synthetic biomarkers provide a powerful tool for early cancer detection, treatment monitoring, and personalized medicine [15, 16, 17, 18]. By integrating principles from chemistry, synthetic biology, and cells engineering, this diagnostic strategy enables the detection of early‐stage tumors by deploying bioengineered sensors in vivo to generate new signals that are either absent or undetectable in biofluids [17, 18]. For instance, S. Bhatia and colleagues developed DNA‐encoded synthetic urine biomarkers that sense tumor‐associated endopeptidases and release CRISPR‐readable DNA barcodes, a method that detected tumors as small as 1–2 mm in diameter in mice [16]. Similarly, Gambhir et al. engineered macrophages to produce luciferase under the control of the arginase‐1 promoter, achieving perfect accuracy (100% sensitivity/specificity) in distinguishing metastatic breast cancer from healthy samples, outperforming conventional biomarkers such as CEA and cfDNA [19]. Despite these advances in early cancer detection, synthetic biomarkers remain largely unexplored for monitoring post‐surgical tumor recurrence. Additionally, the in vivo stability of nucleic acid‐ or polypeptide‐based synthetic biomarkers remains a concern. Thus, developing more robust synthetic biomarkers is crucial for improving early recurrence detection.

Small extracellular vesicles (EVs), also known as exosomes, are nanometer‐sized, phospholipid bilayer‐enclosed vesicles secreted by nearly all cell types [20, 21]. Their lipid bilayer structure facilitates crossing biological membranes while protecting internal bioactive cargo from enzymatic degradation during circulation [22]. Since EVs inherit specific molecular signatures from their parent cells, they are promising diagnostic biomarkers for liquid biopsy, particularly in detecting post‐surgical cancer recurrence [23, 24, 25]. Beyond diagnostics, tumor‐derived EVs play multiple roles in cancer progression, including modulating the immunosuppressive tumor microenvironment [20, 26, 27]. Given their functional diversity, synthetic EVs‐based platforms that integrate diagnostic precision with therapeutic potential could enable early detection and prevention of post‐surgical tumor recurrence.

**FIGURE 1:**
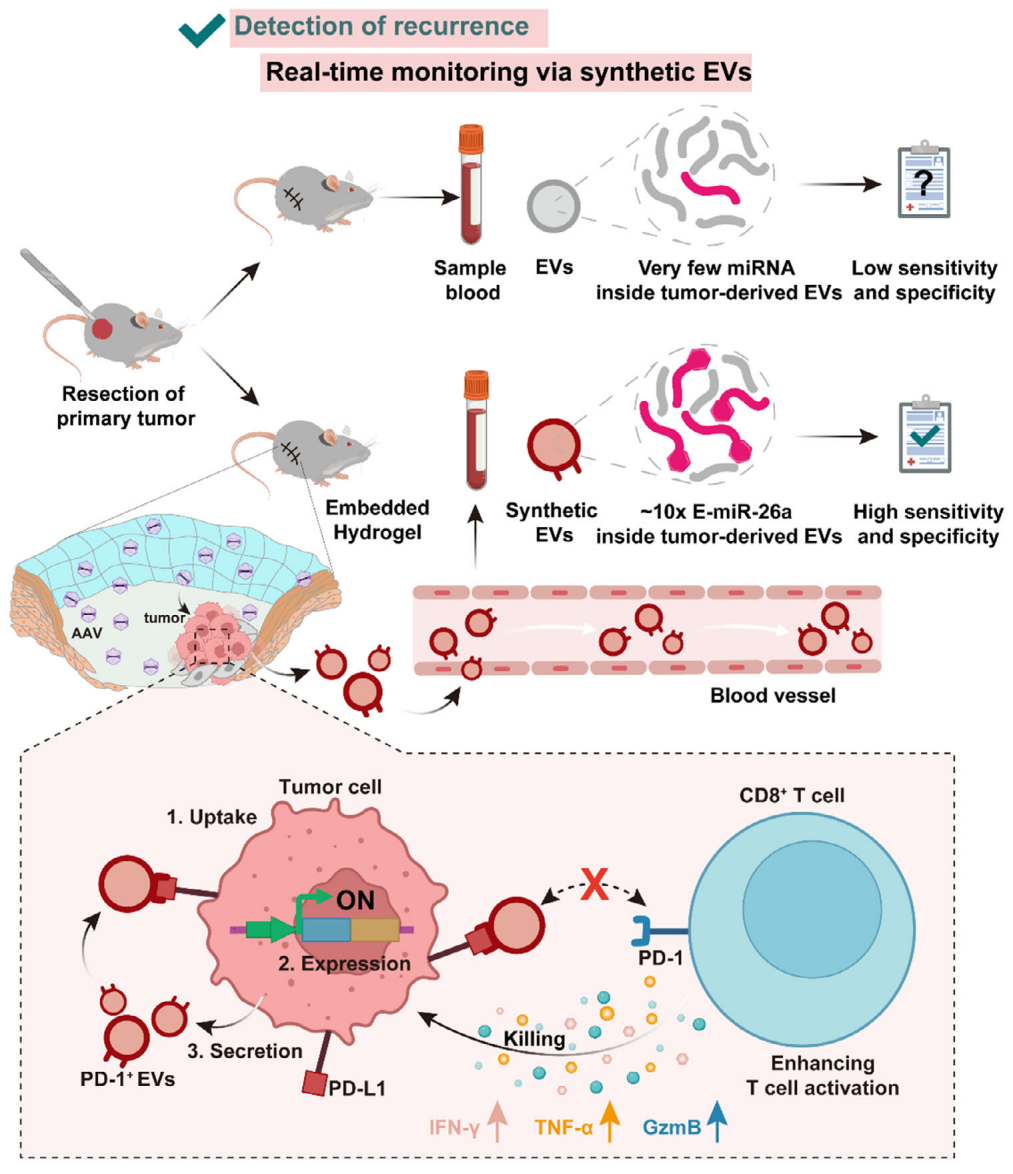
Schematic illustration of synthetic EVs containing E‐miR‐26a or PD‐1 protein for diagnosis and inhibition of post‐surgical cancer recurrence. The hydrogel is implanted at the tumor resection site, which holds gene transfection agents to turn early recurrent tumor cells into synthetic EVs generators. The synthetic EVs are designed to express engineered miR‐26a (E‐miR‐26a) which contains a unique barcoded miRNA sequence, or PD‐1 (a PD‐L1‐blocking agent), thereby enabling early detection and prevention of post‐surgical tumor recurrence.

Here we report an implantable hydrogel system designed to deliver gene transfection agents that selectively reprogram recurrent tumor cells to secrete synthetic EVs, thereby achieving early detection and inhibition of post‐surgical tumor recurrence (Figure 1). Using melanoma as a model, we investigated the biological function of this hydrogel system. Specifically, the synthetic EVs were designed to express engineered miR‐26a (E‐miR‐26a) containing EXOmotifs sorting sequences [28], which enhanced their packaging into EVs and improved detection sensitivity. Furthermore, given that the blockade of the PD‐1/PD‐L1 interaction enhances antitumor immune responses and has demonstrated promising clinical efficacy in certain cancers, especially in melanoma, and that radiotherapy (RT) can upregulate PD‐L1 expression on tumor cells [29], the synthetic EVs were designed to express PD‐1 on their surface, enabling binding to PD‐L1 on both autologous and neighboring tumor cells. This interaction promotes T cells activation, and when combined with RT, effectively suppresses postoperative tumor recurrence. By selectively reprogramming recurrent tumor cells to secrete synthetic EVs with diagnostic and therapeutic functions, this hydrogel system offers a promising strategy for the early detection and effective prevention of post‐surgical cancer recurrence.

## 2 Results

### 2.1 Reprogramming Tumor Cells to Secrete Synthetic EVs for Detection of Tumor Recurrence

**FIGURE 2:**
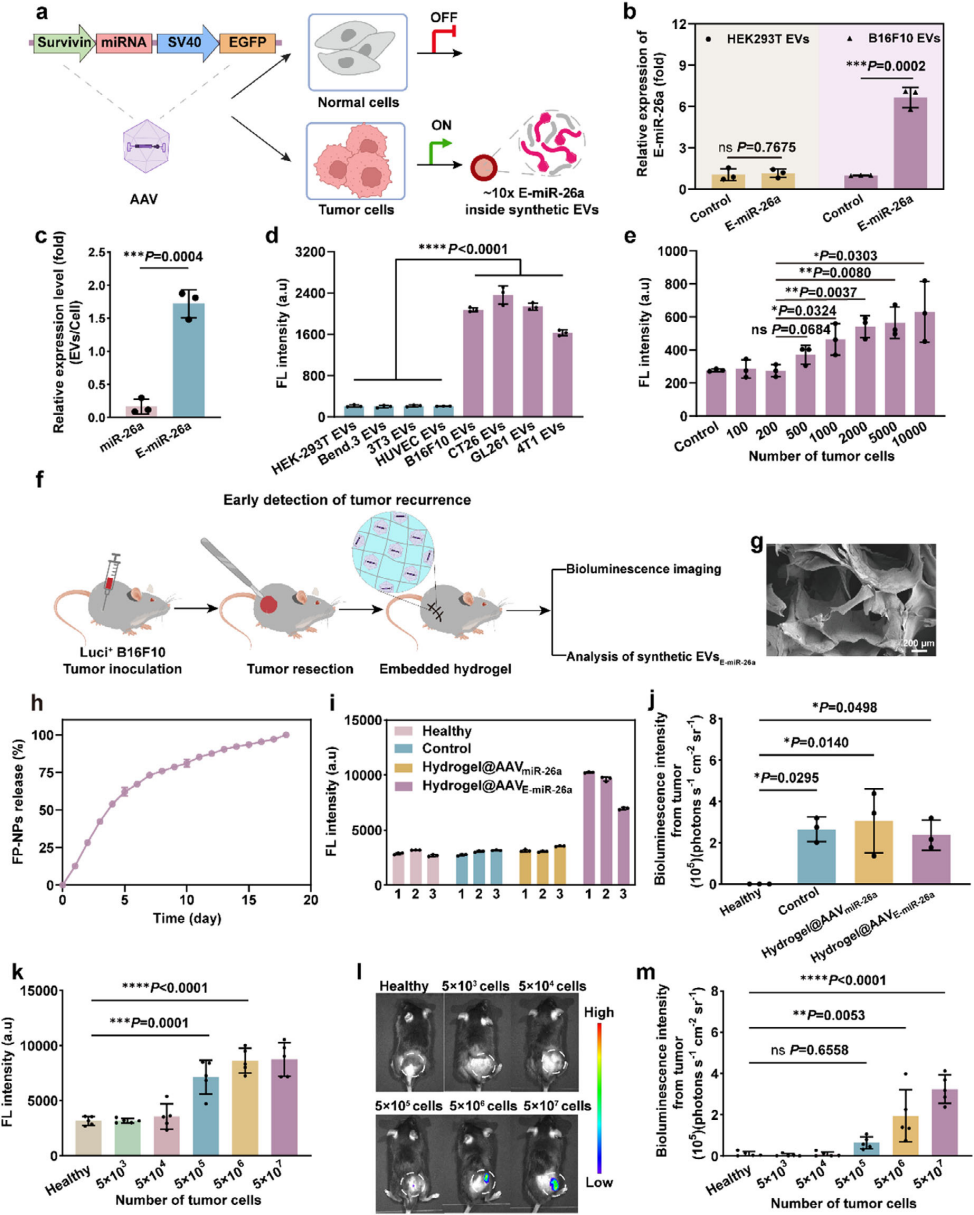
Synthetic EVs for the early detection of post‐surgical tumor recurrence. (a) Schematic illustration of AAV_E‐miR‐26a_ construction with a Survivin promoter‐driven E‐miR‐26a expression system. (b) Relative expression of E‐miR‐26a in EVs derived from B16F10 melanoma cells and HEK293T normal cells. (c) EVs enrichment calculated as the ratio of EVs expression to cellular expression in B16F10 melanoma cells for miR‐26a or E‐miR‐26a containing the EXOmotifs. (d) Level of E‐miR‐26a in EVs from various tumor cells lines (B16F10, CT26, GL261, and 4T1 cells) and normal cell lines (HEK293T, Bend.3, 3T3, and HUVEC cells) following AAV_E‐miR‐26a_ transfection. (e) Level of E‐miR‐26a in EVs from different numbers of B16F10 melanoma cells. (f) Scheme for detecting tumor recurrence. Tumor recurrence was detected through bioluminescence imaging and quantification of E‐miR‐26a in plasma EVs. (g) Representative SEM image of sodium alginate hydrogel. (h) Time‐dependent release profiles of FP‐NPs (size‐comparable to AAV) from sodium alginate hydrogel. (i) Level of E‐miR‐26a in plasma EVs from healthy mice and tumor‐resected mice implanted with blank hydrogel, hydrogel@AAV_miR‐26a_, or hydrogel@AAV_E‐miR‐26a_. (j) Region‐of‐interest analysis of recurrent tumor bioluminescence intensity. (k) Level of E‐miR‐26a in plasma EVs of mice inoculated with different numbers of Luci^+^ B16F10 melanoma cells. (l) Representative images of tumor bioluminescence of mice inoculated with different numbers of Luci^+^ B16F10 melanoma cells. (m) Region‐of‐interest analysis of tumor bioluminescence intensities corresponding to (l).

To selectively reprogram tumor cells to secrete synthetic EVs, we constructed adeno‐associated virus (AAV) vector carrying E‐miR‐26a sequence with a tumor‐responsive Survivin promoter. The Survivin promoter is a widely used tumor‐specific promoter known for its low expression in non‐cancerous cells [30], thereby enhancing tumor‐targeting specificity. The E‐miR‐26a incorporates specific sorting sequences (EXOmotifs, CGGGAG) that facilitate its preferential loading into small EVs [28, 31]; this design promotes efficient packaging and consequently increases the concentration of the detection target (Figure 2a). Thus, AAV_E‐miR‐26a_ could be taken up by both tumor and normal cells, but the tumor‐specific Survivin promoter selectively activates E‐miR‐26a expression only in tumor cells, leading to the secretion of synthetic EVs enriched with E‐miR‐26a. AAV was produced by co‐transfecting HEK293T cells with triple plasmid transfection according to the reported methods [32, 33]. The obtained AAV exhibited a relatively uniform size distribution with an average diameter of 25 nm as anticipated (Figure S1a,b) [34, 35]. Confocal laser scanning microscopy (CLSM) and flow cytometry analysis confirmed successful AAV transfection into B16F10 melanoma cells, with 53.1% of cells expressing EGFP at 72 h (Figure S1c,d).

To evaluate the tumor‐selective reprogramming ability of the produced AAV and the role of EXOmotifs in miRNA secretion, B16F10 cells were transfected with either AAV_E‐miR‐26a_ or AAV_miR‐26a_ (lacking EXOmotifs). EVs isolated from transfected B16F10 cells exhibited no significant differences in morphology, concentration, particle size, zeta potential, or EVs marker protein expression (CD9, CD63, and CD81) compared to control EVs (Figure S2a–e). These results were consistent with previous studies that EVs secreted by engineered cells do not alter the morphology, size, or EVs marker proteins [36, 37, 38]. Notably, RT‐qPCR analysis revealed that E‐miR‐26a expression was significantly elevated in EVs secreted by B16F10 cells compared to the control miR‐26a (Figure S3a,b). In contrast, EVs from HEK293T cells transfected with AAV_E‐miR‐26a_ showed no obvious change in the level of E‐miR‐26a (Figure 2b), confirming the tumor‐specific activity of the Survivin promoter. By calculating the relative abundance of miRNAs in EVs versus cells, we found that E‐miR‐26a was enriched 10‐fold more in EVs than miR‐26a (Figure 2c), indicating that the EXOmotifs significantly enhance the secretion of E‐miR‐26a into EVs. Furthermore, to evaluate the cell specificity of AAV_E‐miR‐26a_, we quantified E‐miR‐26a expression using both RT‐qPCR and a topologically constrained DNA‐mediated one‐pot CRISPR assay we developed previously [39] (detection principle detailed in Figure S4). As shown in Figure 2d and Figure S5a, the expression of E‐miR‐26a within EVs was significantly higher from various tumor cell lines (e.g., B16F10, CT26, GL261, and 4T1 cells) than in normal cell lines. This result indicates that the Survivin promoter drives specific E‐miR‐26a expression in tumor cells, demonstrating the potential tumor specificity of AAV_E‐miR‐26a_ for expressing the target gene. Notably, the sensitivity of our topologically constrained DNA‐mediated one‐pot CRISPR assay was superior to that of RT‐qPCR, detecting EVs secreted by as few as 1000 B16F10 melanoma cells (Figure 2e; Figure S5b). Collectively, these results confirm that the Survivin promoter‐driven AAV vector successfully reprograms tumor cells to selectively express and secrete synthetic EVs enriched with E‐miR‐26a, and that this output could be detected with high sensitivity.

To evaluate the feasibility of using synthetic EVs for detecting post‐surgical tumor recurrence in vivo, we encapsulated the AAV_E‐miR‐26a_ vector in a hydrogel and implanted it at the tumor resection site in tumor‐bearing mice (Figure 2f). The alginate hydrogel, which has already been approved for medical uses [40, 41], was prepared by simply mixing sodium alginate (Alg) and calcium chloride (CaCl_2_) solutions. To investigate the distribution and release behavior of AAV within the hydrogel, fluorescent polystyrene nanoparticles (FP‐NPs), comparable in size to AAV, were used as a surrogate. We examined the impact of different alginate concentrations on hydrogel properties. The results indicated that a 2% alginate concentration achieved optimal performance in terms of encapsulation efficiency and degradation rate (Figure S6a,b). Scanning electron microscopy (SEM) revealed a porous network structure in the 2% alginate hydrogel, while rheological analysis confirmed its elastic property (Figure 2g; Figure S6c). CLSM demonstrated uniform distribution of FP‐NPs within the hydrogel (Figure S6d). Additionally, hydrogel@FP‐NPs exhibited sustained release kinetics in vitro, with a cumulative release of 53.9% by day 4 (Figure 2h). In vivo tracking further confirmed that FP‐NPs within the hydrogel@FP‐NPs remained detectable for two weeks (Figure S6e), indicating that the hydrogel effectively prolonged FP‐NPs retention. Furthermore, the distribution of Cy5‐NHS labeled AAV (AAV/Cy5) within the hydrogel and release behavior of AAV/Cy5 from the hydrogel were also characterized. As shown in Figure S7, AAV/Cy5 was uniformly distributed throughout the hydrogel, and hydrogel system enabled sustained release of AAV/Cy5. The above results suggest that encapsulation in hydrogel is expected to delay the release of AAV at the tumor resection site, allowing prompt response to postoperative recurrent tumors.

To assess the role of hydrogel@AAV_E‐miR‐26a_ in detecting tumor recurrence, we utilized a postoperative residual microtumor model in C57BL/6J mice. Following inoculation with 1 × 10^6^ Luci^+^ B16F10 cells, the primary tumor was surgically resected once it reached a volume of approximately 100 mm^3^. Subsequently, 1 × 10^4^ Luci^+^ B16F10 cells were injected into the surgical site to establish a model of post‐surgical recurrence. Simultaneously, hydrogels containing different components (e.g., blank hydrogel, hydrogel@AAV_miR‐26a_, or hydrogel@AAV_E‐miR‐26a_) were implanted into the resulting surgical cavities. Healthy mice were used as the control group. On day 7 post‐operation, the topologically constrained DNA‐mediated one‐pot CRISPR analysis revealed a significant increase in E‐miR‐26a expression within plasma‐derived EVs in the hydrogel@AAV_E‐miR‐26a_ group compared to the healthy control, blank hydrogel, and hydrogel@AAV_miR‐26a_ group (Figure 2i). Meanwhile, bioluminescence imaging demonstrated a marked increase in signal intensity at the resection site (Figure 2j; Figure S8), confirming tumor recurrence. Collectively, these results indicate that reprogramming tumor cells to secrete synthetic EVs enriched with E‐miR‐26a serves as an effective strategy for reporting tumor recurrence.

To investigate the potential for early tumor detection, we established mouse models with varying tumor burdens by subcutaneously injecting different numbers of Luci^+^ B16F10 cells (ranging from 5 × 10^3^ to 5 × 10^7^ cells). Hydrogel@AAV_E‐miR‐26a_ was subsequently administered locally at the injection site. On day 7, compared to healthy control, the topologically constrained DNA‐mediated one‐pot CRISPR analysis of plasma EVs revealed a significant increase in E‐miR‐26a expression in mice inoculated with 5 × 10^5^ cells (Figure 2k), which is lower than the detection limit of clinical PET imaging (around 10^6^ cells) [42]. Furthermore, the level of E‐miR‐26a in plasma EVs exhibited a positive correlation with the numbers of the initial tumor cell inoculum. These findings were corroborated by in vivo bioluminescence imaging (Figure 2l,m; Figure S9), which demonstrated a direct relationship between tumor burden and the E‐miR‐26a signal intensity derived from plasma EVs. Collectively, these results indicate that synthetic EVs enriched with E‐miR‐26a represent a promising biomarker for the early detection of tumors.

### 2.2 A biodegradable Nanoparticle System for Reprogramming Tumor Cells to Secrete Detectable Synthetic EVs

**FIGURE 3:**
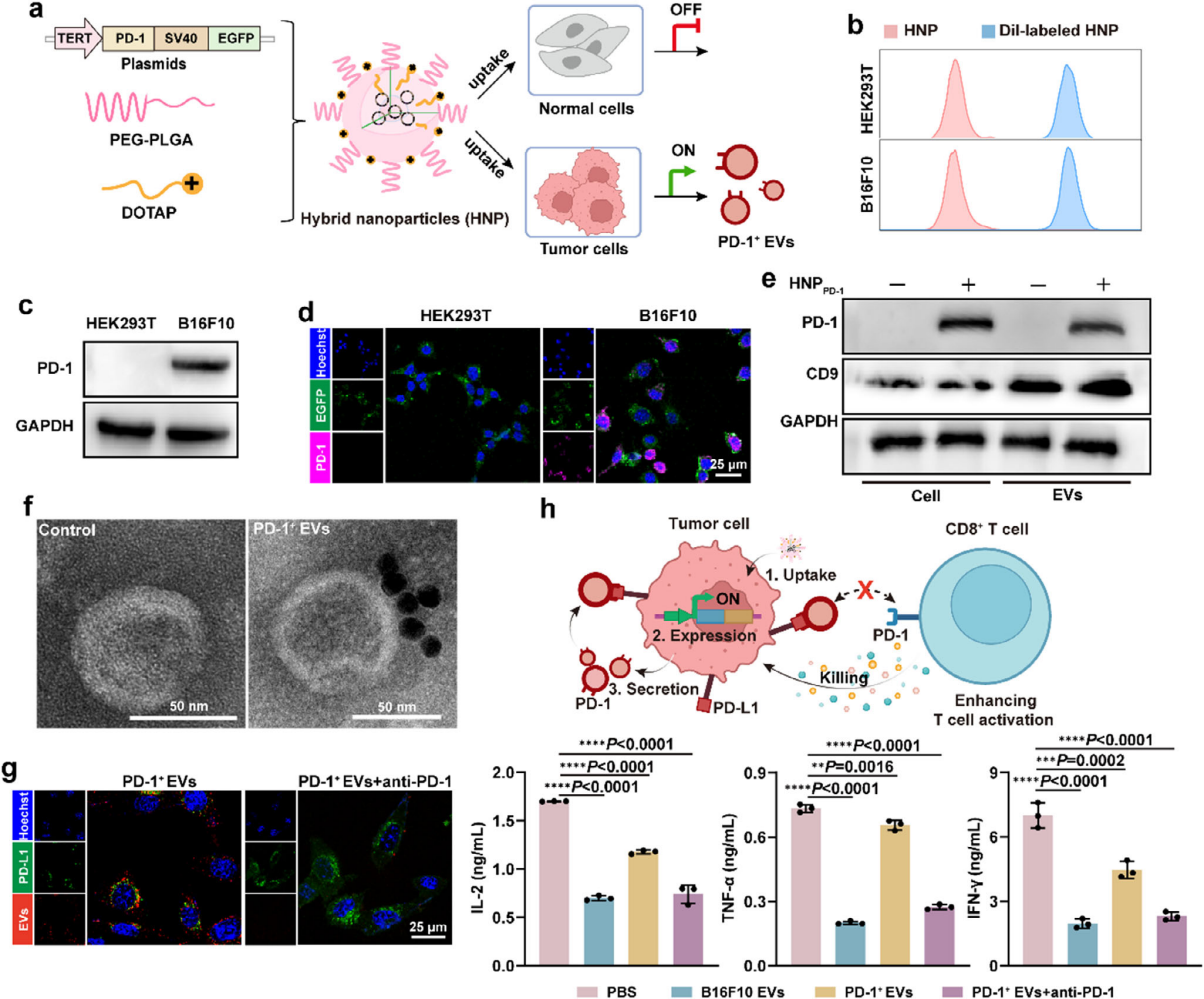
The synthetic PD‐1‐expressing EVs (PD‐1^+^ EVs) enhance the activation of T cells in vitro. (a) Schematic illustration of HNP_PD‐1_ construct. Based on a TERT promoter‐driven PD‐1 expression plasmid, HNP_PD‐1_ delivers the plasmid into both tumor and normal cells, but the tumor‐specific TERT promoter will only turn on in tumor cells, driving the production of PD‐1^+^ EVs. (b) Flow cytometry analysis of cellular uptake. B16F10 melanoma cells and normal HEK293T cells were co‐cultured with DiI‐labeled HNP for 6 h to assess cellular uptake. (c) Western blot analysis of PD‐1 expression in B16F10 and HEK293T cells following transfection with HNP_PD‐1_. (d) Representative CLSM images showing EGFP (green) and PD‐1 (purple) expression in B16F10 and HEK293T cells transfected with HNP_PD‐1_. Nuclei were stained with Hoechst 33342 (blue). PD‐1 was stained with Cy3‐labeled anti‐PD‐1 antibody. (e) Western blot analysis of PD‐1 and CD9 in whole‐cell lysates and EVs derived from both un‐transfected and HNP_PD‐1_‐transfected B16F10 cells. (f) Representative TEM images of EVs immunogold‐labelled with anti‐PD‐1 antibody. PD‐1^+^ EVs were isolated from HNP_PD‐1_‐transfected B16F10 cells, while control EVs were derived from un‐transfected B16F10 cells. (g) CLSM analysis of PD‐1^+^ EVs (with or without anti‐PD‐1 antibody blocking) binding to B16F10 cells. Cells were stained with Cy5/anti‐PD‐L1 antibody (green) and nuclei were stained with Hoechst 33342 (blue). EVs were labeled with CellMask Green (red). (h) Schematic illustration of HNP_PD‐1_‐transfected tumor cells secreting PD‐1^+^ EVs that bind to PD‐L1 on tumor cells, thereby blocking the PD‐1/PD‐L1 pathway to enhance T cells activation. ELISA quantification of IL‐2, TNF‐α, and IFN‐γ levels in the supernatant of anti‐CD3‐stimulated CD8^+^ T cells treated with PBS buffer, B16F10 cell‐derived EVs, PD‐1^+^ EVs, or anti‐PD‐1 antibody blocked PD‐1^+^ EVs.

Despite being a leading platform for therapeutic gene transfer in clinical trials, the widespread application of AAV vectors has been limited by host immune responses against both viral capsid and transgene products [43, 44]. Furthermore, current AAV manufacturing processes are costly, time‐consuming, and not readily amenable to good manufacturing practice standards [45, 46]. To circumvent these limitations of immunogenicity and high production costs, we employed biodegradable nanoparticles as an alternative plasmid delivery system. For this aim, a tumor specific co‐expression plasmid for E‐miR‐26a and EGFP was constructed using Survivin promoter. And the above plasmid was delivered using hybrid nanoparticles (HNP) composed of PEG_5k_‐PLGA_11k_ and the cationic lipid of DOTAP (Figure 3a). TEM revealed no significant morphological changes in the HNP following plasmid encapsulation (Figure S10a). DLS analysis indicated that the average hydrodynamic diameter increased from 92 nm for blank HNP (HNP_Control_) to 108 nm for E‐miR‐26a plasmid‐loaded HNP (HNP_E‐miR‐26a_) (Figure S10b). The zeta potential of HNP_E‐miR‐26a_ also decreased to +29.23 ± 0.41 mV, compared to +32.95 ± 0.73 mV for HNP_Control_, indicating reduction in surface charge due to the incorporation of plasmid (Figure S10c).

We next assessed the cellular uptake of HNP_E‐miR‐26a_ in B16F10 melanoma cells and HEK293T normal cells. After co‐incubation with DiI‐labeled HNP, CLSM analysis demonstrated a time‐dependent increase in uptake (Figure S11a). Flow cytometric analysis confirmed that after 6 h, nearly 100% of cells in both lines were DiI‐positive (Figure 3b), with 60.7% of cells expressing EGFP reporter at 72 h (Figure S11b). EVs isolated from B16F10 cells transfected with HNP_E‐miR‐26a_ exhibited no significant differences in morphology, particle size, or zeta potential, relative to control EVs (Figure S12a–c). However, E‐miR‐26a expression was significantly elevated within EVs secreted by the transfected B16F10 cells (Figure S12d–g), consistent with results achieved via AAV transfection.

Fibrin plays a crucial role in the coagulation cascade, forming the physiological clot essential for wound healing [47]. As a clinically approved biomaterial, fibrin hydrogel is formed through the interaction of fibrinogen and thrombin [48]. Leveraging its proven biocompatibility and functional properties, we developed an in situ‐forming fibrin hydrogel designed to encapsulate HNP_E‐miR‐26a_, facilitate wound healing, and enable the sustained local release of nanoparticles within the tumor resection cavity. Our experiments demonstrated that fibrin hydrogel formed rapidly within 1 min at 37°C after mixing thrombin and fibrinogen (Figure S13a). SEM imaging revealed a porous microstructure in the resulting fibrin hydrogel (Figure S13b). Rheological analysis confirmed the hydrogel's solid‐like behavior, indicated by a storage modulus (*G′*) that consistently exceeded the loss modulus (*G″*) (Figure S13c). We further evaluated the degradation kinetics of the fibrin hydrogel, finding moderate degradation under physiological conditions (pH 7.4) and significantly accelerated breakdown in an acidic microenvironment (pH 6.5) (Figure S13d). CLSM confirmed the uniform distribution of DiI‐labeled HNP within the fibrin matrix (Figure S13e). In vivo fluorescence tracking demonstrated that the hydrogel encapsulation prolonged the retention of DiI‐labeled HNP, which remained detectable at the site for up to 20 days (Figure S13f).

We next evaluated the ability of hydrogel@HNP_E‐miR‐26a_ to detect tumor recurrence in vivo (Figure S14a). Following primary tumor resection, hydrogels loaded with different components (e.g., blank hydrogel, hydrogel@HNP_miR‐26a_, or hydrogel@HNP_E‐miR‐26a_) were implanted into the surgical cavities. Tumor recurrence was monitored via bioluminescence imaging, and miRNA levels were quantified in both tumor tissues and plasma EVs. By day 7, all groups exhibited significantly stronger and more homogeneous bioluminescence signals, confirming tumor recurrence (Figure S14b). Quantitative analysis revealed that miR‐26a expression in tumor tissues was 5‐ to 8‐fold higher in the hydrogel@HNP_miR‐26a_ group compared to the blank hydrogel, while E‐miR‐26a levels in the hydrogel@HNP_E‐miR‐26a _group were 4‐ to 6‐fold higher (Figure S14c). Notably, E‐miR‐26a expression in plasma EVs was markedly elevated in the hydrogel@HNP_E‐miR‐26a_ group relative to both the blank hydrogel and hydrogel@HNP_miR‐26a_ groups (Figure S14d). miR‐26a showed high expression in tumor tissues but low levels in plasma EVs, suggesting that it was largely retained within tumor cells, leading to its accumulation locally. In contrast, the incorporation of EXOmotifs promoted selective secretion of E‐miR‐26a into EVs, enabling tumor cells to release E‐miR‐26a‐positive EVs into the circulation and consequently increase the concentration of the detection target, thereby enhancing the sensitivity of tumor recurrence detection.

### 2.3 Synthetic PD‐1^+^ EVs act as Competitive Antagonists to Reverse T Cells Suppression

Following the timely detection of tumor recurrence, effective therapy is critical to enhance patient survival. Notably, the hydrogel system containing transfection agents to reprogram tumor cells into synthetic EVs generators, as presented in this study, could be expanded as a therapeutic strategy. The blockade of the PD‐1/PD‐L1 interaction enhances antitumor immune responses and has demonstrated promising clinical efficacy in certain cancers [49, 50]. However, the widespread application of conventional antibody‐based inhibitors is often limited by their complex manufacturing, purification, and high production costs, which impose significant financial burdens on patients [51]. To overcome this limitation, we embedded a synthetic EVs generation system at the tumor resection site, which reprograms tumor cells to secrete PD‐1^+^ EVs using telomerase reverse transcriptase (TERT) promoter (Figure 3a). These engineered EVs act as competitive antagonists for PD‐L1, effectively blocking the immunosuppressive interaction with both autologous and neighboring tumor cells to restore antitumor immune responses. Specifically, CLSM confirmed robust EGFP reporter expression in both B16F10 and HEK293T cells post‐transfection (Figure 3d), while the TERT promoter selectively induced PD‐1 expression in B16F10 cells (Figure 3c,d). Western blot revealed the presence of PD‐1 in both HNP_PD‐1_‐transfected B16F10 cells and their EVs (Figure 3e). Immuno‐electron microscopy further demonstrated that PD‐1^+^ EVs exhibited a membrane topology similar to that of T cells surface PD‐1, with the extracellular domain exposed on the EVs surface (Figure 3f). Functional validation revealed efficient binding of CellMask Green‐labeled PD‐1^+^ EVs to PD‐L1 (labeled with anti‐PD‐L1‐Cy5) on B16F10 cell surfaces (Figure 3g). Since the aberrant overexpression of PD‐L1 proteins in tumor cells such as melanoma, PD‐1^+^ EVs may confer the enhanced tumor‐binding ability [37]. Importantly, these PD‐1^+^ EVs effectively competed for PD‐L1 binding on T cells, thereby restoring the antitumor activity of CD8^+^ T cells (Figure 3h). Consistent with this functional rescue, enzyme‐linked immunosorbent assay (ELISA) quantification of CD8^+^ T cells cytokines revealed that while EVs from parental B16F10 cells suppressed the production of IL‐2, TNF‐α, and IFN‐γ, PD‐1^+^ EVs significantly reversed this immunosuppressive effect. Pre‐incubation of PD‐1^+^ EVs with anti‐PD‐1 antibody nearly abolished this reversal. Collectively, these results indicate that synthetic PD‐1^+^ EVs enhance T cells activation in vitro.

### 2.4 Synergistic Anti‐Tumor Recurrence Combining RT and Synthetic PD‐1^+^ EVs Secretion

**FIGURE 4:**
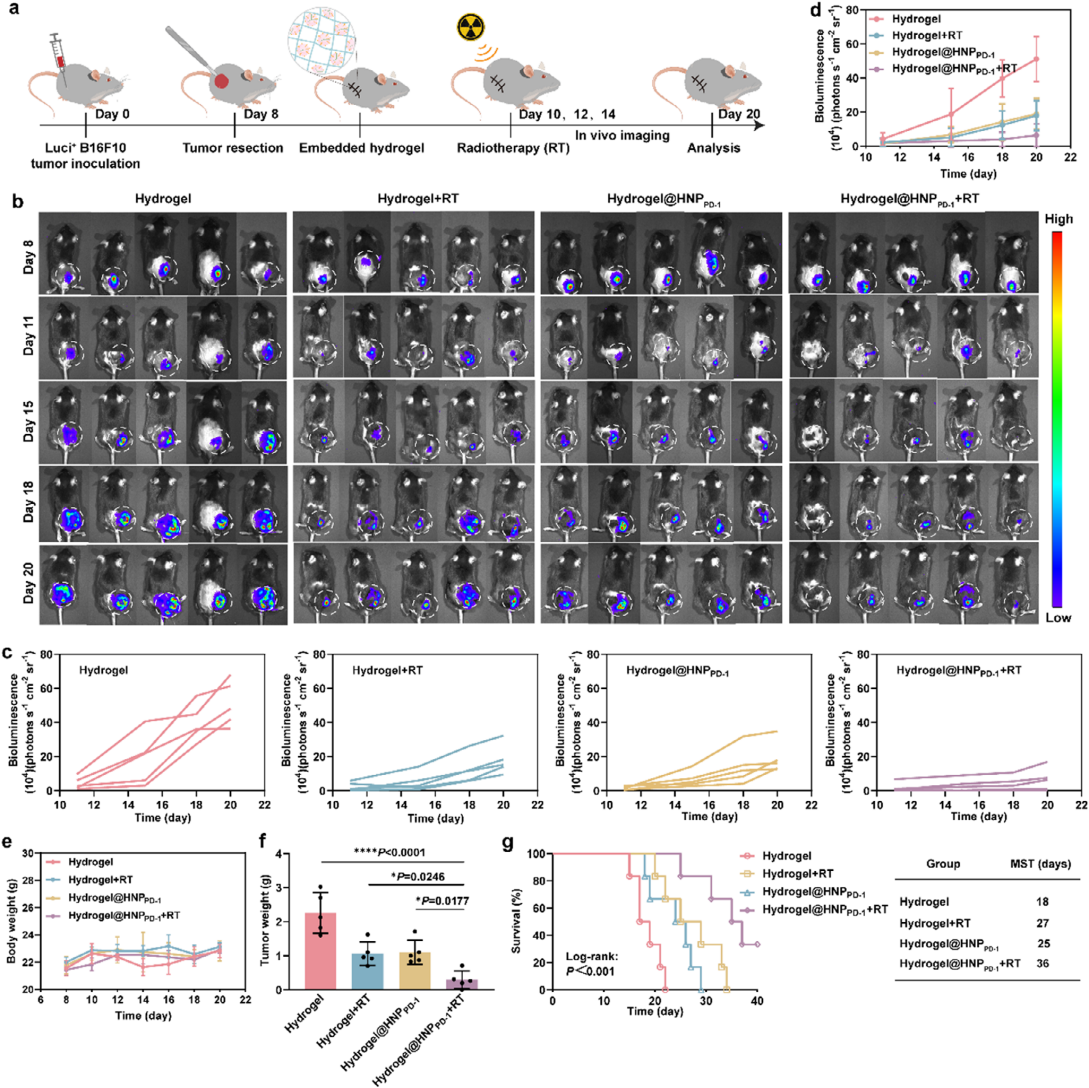
Localized synthetic PD‐1^+^ EVs combined with RT prevents postoperative melanoma recurrence. (a) Schedule of the experimental design. (b) In vivo bioluminescence imaging of B16F10 melanoma‐bearing mice after resection of the primary tumor at different time periods (n = 5 mice per group). Images associated with day 8 were taken before surgery. (c) Individual and (d) average tumor growth kinetics in different groups. (e) Weight changes of mice in different groups. (f) Final tumor weights of the B16F10 melanoma‐bearing mice after the treatments. (g) Survival curves of the B16F10 melanoma‐bearing mice treated with different groups (n = 5 mice per group). The MST of each group was provided.

Immune checkpoint therapy combined with RT represents a promising anti‐tumor treatment strategy [52], particularly for preventing post‐surgical cancer recurrence [2, 33]. RT exerts multiple immune‐stimulating effects, such as the release of tumor‐associated antigens and damage‐associated molecular patterns, which collectively promote immune cell priming and disrupt the immunosuppressive tumor stroma [53]. Encouraged by the superior ability of synthetic PD‐1^+^ EVs to enhance T cells activation in vitro, we next evaluated their therapeutic efficacy in combination with RT using a post‐surgical tumor recurrence model (Figure 4a). Following the surgical resection of established Luci^+^ B16F10 tumors on day 8 post‐inoculation, a recurrence model was established by simultaneously injecting 1 × 10^4^ Luci^+^ B16F10 cells. The above mice were then randomized into four treatment groups: hydrogel (control), hydrogel + RT (6 Gy, 3 cycles), hydrogel@HNP_PD‐1_, and hydrogel@HNP_PD‐1_ + RT (6 Gy, 3 cycles). Bioluminescence imaging revealed no significant tumor growth suppression in the hydrogel‐only group (Figure 4b–d). In contrast, both hydrogel + RT and hydrogel@HNP_PD‐1_ monotherapies inhibited melanoma progression, achieving tumor growth inhibition rates of 53.0% and 51.0%, respectively. Notably, the combination of hydrogel@HNP_PD‐1_ with RT demonstrated a synergistic antitumor effect, yielding an 87% inhibition rate. At the treatment endpoint, tumor weights in the combination group were only 13.2% of those in the control group, a significant enhancement compared to the hydrogel + RT (47.0%) or hydrogel@HNP_PD‐1_ alone (48.8%) groups (Figure 4f).

We further assessed the effects of each treatment on tumor cell proliferation and apoptosis. The hydrogel@HNP_PD‐1_ + RT group exhibited a significantly lower proportion of Ki‐67‐positive cells, indicating potent inhibition of proliferation (Figure S15). Histological analysis of hematoxylin and eosin (H&E)‐stained sections revealed extensive tumor necrosis, and terminal‐deoxynucleotidyl transferase mediated nick end labeling (TUNEL) staining confirmed a substantial increase in apoptotic cells (Figure S15). To further validate the anti‐recurrence efficacy, we monitored survival. Both monotherapy regimens significantly prolonged overall survival, with the combination group exhibiting the longest survival (Figure 4g). The median survival time (MST) of mice receiving hydrogel@HNP_PD‐1_ + RT was extended to 36 days, surpassing that of the hydrogel + RT (27 days) and hydrogel@HNP_PD‐1_ (25 days) groups.

Furthermore, material biocompatibility and systemic safety were assessed by monitoring mouse body weight and performing histopathological analyses. No significant body weight loss was observed throughout the treatment period; instead, a slight increase was noted (Figure 4e). Histopathological examination of major organs revealed no apparent abnormalities or lesions in any treatment groups (Figure S16a). Complete blood count analysis showed mice receiving RT (hydrogel + RT and hydrogel@HNP_PD‐1_ + RT) groups exhibited a modest reduction in white blood cell (WBC) and platelet (PLT) counts following treatment (Figure S16b). This decrease may be attributed to radiation‐induced suppression of bone marrow hematopoietic function, a well‐documented side effect in both preclinical and clinical settings [54, 55]. Collectively, these results demonstrate that the hydrogel@HNP_PD‐1_ formulation itself possesses a favorable safety profile, with no significant systemic toxicity or treatment‐related adverse events observed.

### 2.5 Combination Therapy Reverses Immunosuppression by Enhancing T Cells Infiltration and Activation

**FIGURE 5:**
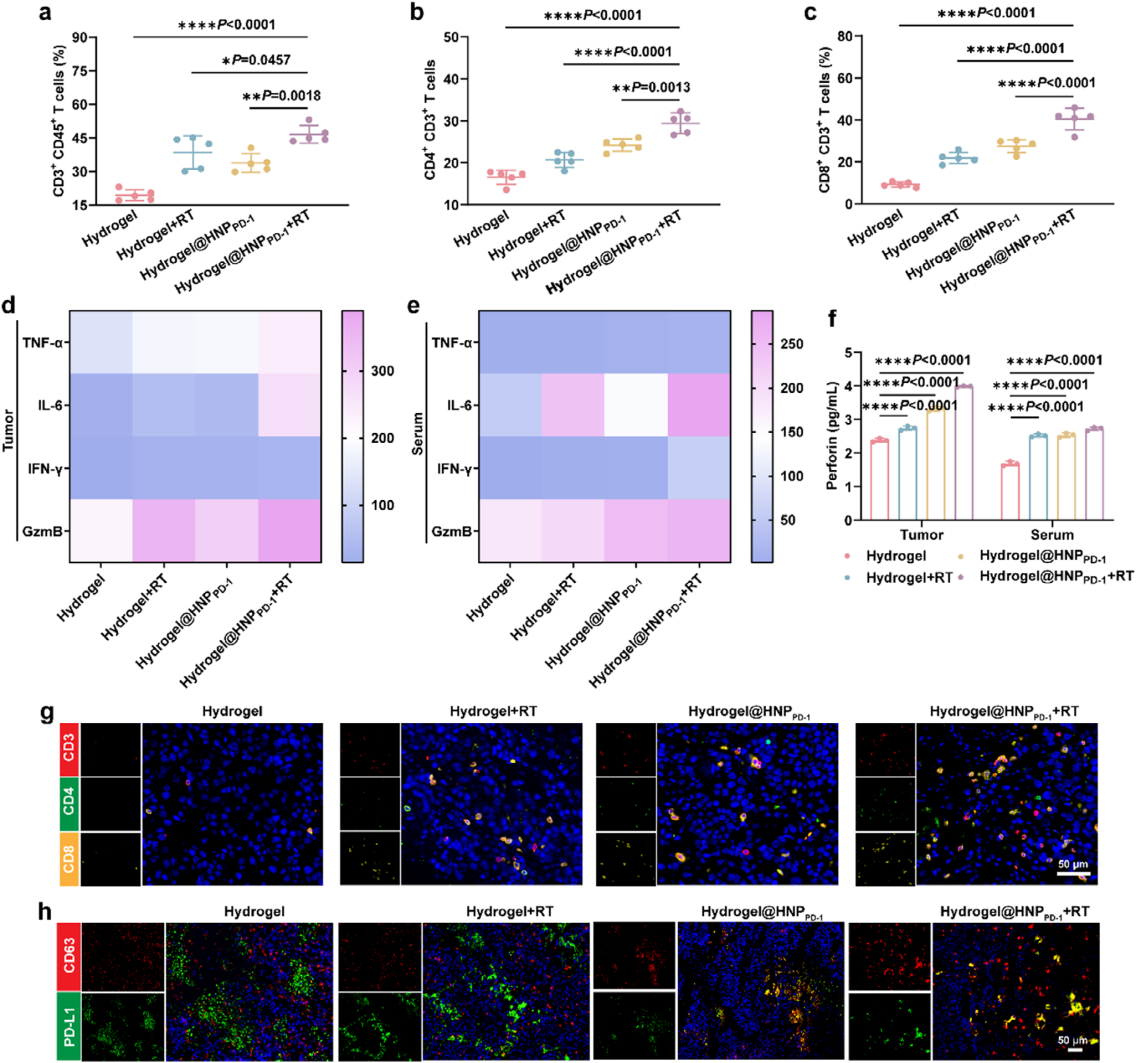
Enhancing intra‐tumoral T cells infiltration and activation via synthetic PD‐1^+^ EVs combination with RT. (a‐c) Representative flow cytometric quantification of (a) CD3^+^ CD45^+^ T cells, (b) CD4^+^ CD3^+^ T cells, and (c) CD8^+^ CD3^+^ T cells within tumors following various treatments. (d, e) ELISA measurement of cytokines (such as TNF‐α, IL‐6, IFN‐γ, and GzmB) levels in the (d) tumor and (e) serum from B16F10 melanoma‐bearing mice after different treatments. (f) ELISA measurement of perforin levels in the tumor and serum from B16F10 melanoma‐bearing mice after different treatments. (g) Representative immunofluorescence images of tumors from different groups (red: CD3^+^ T cells, green: CD4^+^ T cells, and brownish yellow: CD8^+^ T cells). Scale bar, 50 µm. (h) Representative immunofluorescence images of tumors from different groups (red: CD63^+^ EVs, green: PD‐L1^+^ tumor cells). Scale bar, 50 µm.

To investigate whether the enhanced therapeutic efficacy of hydrogel@HNP_PD‐1_ was mediated by improved intra‐tumoral T cells infiltration and activation, we analyzed the population and phenotypic characteristics of T cells in tumor tissues following various treatments. Mice treated with hydrogel@HNP_PD‐1_ +RT exhibited a 3.05‐fold increase in the proportion of CD3^+^ T cells compared to those receiving hydrogel alone (Figure 5a; Figure S17). Further analysis revealed that the hydrogel@HNP_PD‐1_ +RT group displayed 2.38 and 5.98‐fold increases in tumor‐infiltrating CD3^+^CD4^+^ T cells and CD3^+^CD8^+^ T cells, respectively, relative to the hydrogel control (Figure 5b,c; Figure S17), indicating robustly enhanced T cells infiltration. Immunofluorescence staining of tumor sections also confirmed these findings, demonstrating high infiltration of CD8^+^ and CD4^+^ T cells in the hydrogel@HNP_PD‐1_ + RT group, in contrast to the minimal infiltration observed in the hydrogel‐treated group (Figure 5g). Subsequently, we assessed the activation status of T cells via flow cytometry. The population of granzyme B (GzmB)^+^CD8^+^ T cells was 1.66 and 1.41‐fold larger in the hydrogel@HNP_PD‐1_ + RT group than in the RT‐alone and hydrogel@HNP_PD‐1 _groups, respectively (Figure S18). Consistent with enhanced T cells activation, ELISA analysis of the tumor microenvironment showed elevated levels of TNF‐α, IL‐6, IFN‐γ, GzmB, and perforin in the hydrogel@HNP_PD‐1_ + RT group (Figure 5d,f). Similar trends were observed in serum samples (Figure 5e,f). Collectively, these results demonstrate that the combination of hydrogel@HNP_PD‐1_ and RT potently enhances T cells infiltration and activation, thereby contributing to its significant antitumor efficacy.

To determine whether these effects were attributable to interactions between synthetic PD‐1^+^ EVs and tumor cells, we performed co‐staining with tumor cells (an anti‐PD‐L1 antibody, green) and EVs marker protein CD63 (anti‐CD63 antibody, red). As shown in Figure 5h, binding of EVs to PD‐L1 on tumor cells was significantly increased in mice treated with hydrogel@HNP_PD‐1_ and hydrogel@HNP_PD‐1_ + RT compared to hydrogel alone. This observation was likely due to the specific binding of synthetic PD‐1^+^ EVs to PD‐L1 expressed on tumor cells. Collectively, these results demonstrate that HNP_PD‐1_ successfully induced the secretion of synthetic PD‐1^+^ EVs, and that its combination with RT effectively reversed the immunosuppressive tumor microenvironment, leading to robust cytotoxic T cells infiltration and activation.

## 3 Discussion

Bioengineered sensors deployed in vivo to generate molecular reporters within diseased microenvironments are reshaping the landscape of liquid biopsy, owing to their high specificity, sensitivity, and controllability [15, 16, 18, 19, 56]. Preclinical studies have demonstrated that synthetic biomarkers based on tumor‐specific enzyme activity could achieve the low limit of detection required for early disease detection [16, 19, 57]. For instance, Bhatia et al. developed a sensor, composed of nanoworm nanoparticles conjugated with mass‐barcoded peptide substrates, which successfully discriminated colorectal tumors with volumes 60% smaller than those detectable by the shed serum biomarker CEA [57]. Despite these advances, the clinical translation of polypeptide‐based synthetic biomarkers is hindered by poor in vivo stability, as free peptides are typically subject to rapid renal clearance. To address this limitation, building upon the remarkable stability in circulation of EVs and protecting their cargo from degradation [58, 59, 60, 61], we developed a strategy that reprograms recurrent tumor cells to secrete synthetic EVs carrying E‐miR‐26a. By incorporating specific EXOmotifs sorting sequences into E‐miR‐26a, we enhanced its secretion into EVs, thereby improving the sensitivity for detecting recurrent tumors. Our results demonstrated an approximately 10‐fold increase in E‐miR‐26a concentration within EVs, enabling detection of tumors at an early stage in mice with as few as 5 × 10^5^ tumor cells. Moreover, we compared the currently available methods, including MRI, CT, circulating tumor cells (CTC), and circulating tumor DNA (ctDNA) with this work, the advantages and disadvantages were listed in Table S2. This approach overcomes the sensitivity and specificity limitations of endogenous biomarkers, allowing for the reliable detection of tumor recurrence‐specific signals even at low concentrations.

Following the timely detection of tumor recurrence, effective therapeutic strategies are imperative to improve the survival period of patients. Tumor‐derived EVs have been demonstrated to play multiple roles in cancer progression, particularly by modulating immune responses [20, 26, 27]. For instance, melanoma‐derived EVs expressing PD‐L1 inhibit the antitumor activity of CD8^+^ T cells [62], and similarly, induce T cells exhaustion in tumor‐draining lymph nodes to accelerate metastatic growth [63]. To counteract this mechanism, we developed an implantable, in situ synthetic EVs generation system that reprograms tumor cells at the resection site to secrete PD‐1^+^ EVs. These engineered EVs act as competitive PD‐L1 antagonists, disrupting immunosuppressive interactions in autologous and neighboring tumor cells to restore anti‐tumor immunity. Our results confirm their high binding specificity for PD‐L1 on tumor cells. Unlike current approaches that primarily engineer cells in vitro to secrete PD‐1^+^ EVs for subsequent intravenous or intraperitoneal injection [36, 37], our approach directly reprograms tumor cells in situ to continuously produce PD‐1^+^ EVs at the recurrence site in mice. This localized delivery maximizes therapeutic effects while minimizing systemic exposure and side effects. Furthermore, when combined with RT, these synthetic PD‐1^+^ EVs synergistically enhance T cells activation and significantly improve antitumor efficacy.

Although synthetic EVs offer a promising strategy for the early detection and inhibition of post‐surgical cancer recurrence, future studies should further explore their diagnostic and therapeutic potential across a broader range of solid tumor models and in clinical trials. Controlling the specificity of gene expression and minimizing off‐target effects are critically important in the development of AAV‐based clinical application. To achieve selective reprogramming of tumor cells to secrete synthetic EVs, we employed Survivin and TERT promoter, which are highly active in most cancer cells but remain tightly silenced in somatic cells. For melanoma‐specific applications, the use of the tyrosinase promoter should also be investigated to enhance cell‐type specificity [50]. Specificity also can be achieved by using cell type‐specific promoters to restrict ectopic expression of transgenes, engineering AAV capsids to enhance cell type tropism, and use of tissue‐specific promoters [64]. Furthermore, before clinical translation, their immunogenicity, metabolic fate, long‐term biodistribution, and ethical considerations must be thoroughly evaluated and addressed prior to any clinical in vivo application [65]. Additionally, future research should establish standardized quantitative benchmarks, including detection limits, response kinetics, and dynamic ranges, that reliably correlate E‐miR‐26a^+^ EVs signal outputs with disease progression.

## 4 Conclusion

In summary, this study successfully developed an implantable post‐surgical hydrogel system that specifically responds to recurrent tumor cells and reprograms them to secrete synthetic EVs with ‘diagnostic/therapeutic’ functions. The system enables the detection of postoperative tumor recurrence and effectively inhibits the growth of recurrent tumors. This innovative approach provides a promising strategy for post‐operative management, potentially enabling both early recurrence detection and therapeutic intervention in a single platform.

## 5 Experimental Section

### 5.1 Materials

The plasmid was obtained from Yunzhou Biotechnology Co., Ltd. (Guangzhou, China). Plasmid maps of miR‐26a, E‐miR‐26a, and PD‐1 (encoded by the Pdcd1 gene) was shown in Figure S19. Thrombin was acquired from Shanghai Yuanye Bio‐Technology Co., Ltd. (Shanghai, China). Fibrinogen was purchased from Beijing Solarbio Life Sciences Co., Ltd. (Beijing, China). PEG_5K_‐PLGA_11K_ and (2,3‐dioleoyloxy‐propyl)‐trimethylammonium (DOTAP) were purchased from Xi'an Ruixi Biotechnology Co., Ltd. (Xi'an, China). Anti‐CD9, anti‐CD63, and anti‐CD81 antibodies were obtained from Abcam P.L.C. (Cambridge, England). Anti‐PD‐L1/Cy5 and anti‐PD‐1/Cy3 antibodies were acquired from Bioss Antibodies (Beijing, China). CD8^+^ T cells selection kit was acquired from BioLegend, Inc. (California, USA). ELISA kits were obtained from Lianke Biotechnology Co., Ltd. (Hangzhou, China).

### 5.2 Collection and Purification of AAV

A three‐plasmid system, comprising a Rep‐Cap expression plasmid, helper plasmid expressing adenoviral genes (E4, E2A, and VA), and a transfer plasmid carrying the target gene, was utilized for the AAV packaging. The relevant plasmid sequences were provided in Table S1. The plasmids were co‐transfected into HEK293T cells to produce AAV particles. The AAV was harvested and purified using a commercial GENMED AAV precipitation purification kit (Shanghai Jiemai Gene Medical Technology Co., Ltd., China). Briefly, 72 h post‐transfection, the cell pellet was harvested and lysed with 1 mL GENMED lysis solution via vortex for 30 s, followed by incubation at room temperature for 5 min. The lysate was centrifuged at 10 000 g for 5 min at 4°C, and the supernatant was collected. Then, 100 µL of GENMED precipitation solution was added to the supernatant, followed by vortexing for 60 s. The mixture was applied to a GENMED high‐purity column and centrifuged at 7500 g for 5 min at 4°C to obtain the purified AAV suspension.

### 5.3 Characterization of AAV

The morphology of AAV particles was observed using a biological TEM (L120CG2, Thermo Scientific/Talos). For this analysis, a 10 µL aliquot of diluted AAV was dropped to a copper grid, stained with a 2% phosphotungstic acid solution, and imaged at an accelerating voltage of 120 kV. Subsequently, the hydrodynamic particle size and zeta potential of the AAV were measured by DLS using a Nano ZS‐90 instrument (Malvern Instruments).

### 5.4 AAV Transfection and Expression

The expression of EGFP in AAV transfected cells was evaluated using both CLSM and flow cytometry. For this purpose, B16F10 cells were seeded into 24‐well plates and incubated with the AAV at a suitable multiplicity of infection for 24–72 h. For CLSM imaging, cells were fixed with 4% paraformaldehyde and nuclei were stained with Hoechst 33342. Samples were then imaged using CLSM. For flow cytometric analysis, cells were harvested and resuspended in 100 µL of phosphate‐buffered saline (PBS), and the proportion of EGFP‐positive cells was quantified by flow cytometry.

### 5.5 Extraction of EVs

Exosomes were isolated from the collected cell supernatants by differential centrifugation techniques in accordance with previously reported method [66, 67]. The collected cell supernatants were subjected to sequential centrifugation at 500 g, 4°C for 10 min, 2000 g, 4°C for 10 min, and 10 000 g, 4°C for 30 min to remove cellular debris and large vesicles, respectively. The supernatants were then filtered through a 0.22 µm membrane to eliminate dead cells and residual debris. Following this, the supernatants were ultracentrifuged at 110 000 g, 4°C for 2 h to obtain an EVs pellet. Using this identical protocol, EVs from cells transfected with AAV or HNP nanoparticles were extracted. The morphology of EVs was observed using TEM, and the content of miRNA‐26a or E‐miRNA‐26a within the EVs was analyzed using RT‐qPCR analysis.

### 5.6 Characterization of EVs

Nanoparticle tracking analysis: The concentration of EVs was measured using NTA with a NanoSight NS300 instrument (Malvern Instruments) equipped with a Blue 488 laser and sCMOS camera, following the manufacturer's standard protocol. Briefly, exosomes were diluted to an appropriate concentration in PBS and loaded into a syringe for analysis. For each sample, three 30 s videos were recorded under the following settings: temperature: 25°C, camera level: 12, number of frames: 749, viscosity: (water) 0.868–0.870 cP, slider shutter: 1232, slider gain: 219 and frames per second (FPS): 25. The recorded videos were analyzed using NanoSight NTA Software v3.2 Build 3.2.16 to determine particle size and concentration.

Western blotting analysis: Cells and exosomes were lysed using RIPA lysis buffer, and total proteins were extracted by centrifugation [68]. The total protein concentration of the samples was determined using BCA protein quantification kit. Proteins were separated by 10% SDS‐PAGE and transferred onto PVDF membranes using an electrophoresis and transfer system. The membranes were incubated overnight at 4°C with the following primary antibodies: anti‐CD9, anti‐CD63, anti‐CD81, anti‐PD‐1, and anti‐GAPDH. Subsequently, membranes were incubated with the corresponding secondary antibody at 4°C for 1 h and visualized using chemiluminescence detection system (GelView6000 ProII, BLT Photon Technology, China).

### 5.7 Preparation and Characterization of Sodium Alginate Hydrogel

Sodium alginate solutions at concentrations of 0.5%, 1.0%, and 2.0% were prepared, along with CaCl_2_ solution at concentration of 100 mg/mL. These sodium alginate solutions were then mixed with the CaCl_2_ solution at a 1:1 volume ratio to form hydrogels of three distinct concentrations. Rheological tests of hydrogels were analyzed using a rheometer (DHR‐2, TA, USA). To evaluate degradation behavior, hydrogels were incubated in PBS buffer at 37°C, and their mass loss was measured at predetermined time intervals. The cross‐sectional morphology of the hydrogel prepared with 2% sodium alginate was examined using SEM (TM4000Plus, Hitachi, Japan).

### 5.8 Evaluation of the Distribution and Release of AAV in Hydrogels

To investigate the distribution and release behavior of AAV within the hydrogel, FP‐NPs of comparable size were employed as a surrogate. An alginate hydrogel was formed by homogeneously dispersing FP‐NPs into a sodium alginate solution, followed by cross‐linking with CaCl_2_. The spatial distribution of the encapsulated FP‐NPs was subsequently analyzed using CLSM. For the in vitro release study, FP‐NPs‐encapsulating hydrogel (Gel@FP‐NPs) were immersed in PBS buffer at 37°C. The released FP‐NPs were measured at predetermined intervals using a multifunctional microplate reader. To evaluate the in vivo sustained‐release profile, mice were divided into two groups following dorsal hair removal. One group received a subcutaneous injection of free FP‐NPs, while the other was implanted with Gel@FP‐NPs. Fluorescence signal intensity at the implantation site was monitored over time using an IVIS spectral imaging system (Lumina XRMS Series III, PerkinElmer, USA).

To characterize the release behavior of AAV from the hydrogel, AAV/Cy5 was prepared. Specifically, 250 nM Cy5‐NHS dye was dissolved in Na_2_CO_3_/NaHCO_3_ buffer solution (pH 9.3), followed by the addition of the AAV vector and incubation at room temperature for 2 h. The mixture was then transferred to a 50 kDa ultrafiltration tube and centrifuged at 7000 rpm for 15 min to remove free Cy5‐NHS and obtain AAV/Cy5. Next, 40 µL of AAV/Cy5 was mixed with 160 µL of 20 mg/mL sodium alginate solution, and 200 µL of 100 mg/mL CaCl_2_ solution was added to form hydrogel@AAV/Cy5. The hydrogel was sliced and imaged using CLSM to observe the distribution of AAV/Cy5 within the hydrogel. Furthermore, the hydrogel@AAV/Cy5 was immersed in PBS buffer and incubated in a constant temperature shaking incubator at 37°C. At designated time points, aliquots of the release medium were collected to measure fluorescence intensity, and an equal volume of fresh release medium was immediately added to maintain a constant volume.  The release amounts of AAV at different time points were calculated based on the fluorescence intensity values.

### 5.9 Preparation and Characterization of HNP Nanoparticles

The HNP nanoparticles were prepared using the double emulsion solvent evaporation method. Briefly, the plasmid was emulsified in 500 µL of chloroform containing 1 mg of DOTAP and 25 mg of PEG_5k_‐PLGA_11k_ using an ultrasonic processor for 1 min in an ice bath. This primary emulsion was then added to 5 mL of DNase/RNase‐free water and further emulsified to form the final water‐oil‐water emulsion. Subsequently, chloroform was evaporated using a rotary evaporator, obtaining the HNP nanoparticles. Using this identical protocol, HNP _control_, HNP _miR‐26a_, HNP _E‐miR‐26a_, and HNP _PD‐1_ were prepared. The morphology of the HNP nanoparticles was characterized using a TEM, while their hydrodynamic diameter and zeta potential were measured using DLS.

### 5.10 Cellular Uptake and EGFP Expression of HNP Nanoparticles

The cellular uptake of HNP nanoparticles was evaluated using CLSM and flow cytometry. For CLSM analysis, B16F10 cells were seeded into 24‐well plates and incubated with DiI‐labeled HNP nanoparticles for 0, 2, 4, 6, and 8 h. Subsequently, the cells were washed with PBS buffer to remove free nanoparticles, fixed with 4% paraformaldehyde, and the nuclei were stained with Hoechst 33342 prior to imaging. For flow cytometry analysis, B16F10 and HEK293T cells were incubated with DiI‐labeled HNPs for 6 h. The cells were then harvested by trypsinization, washed, resuspended in 100 µL of PBS, and analyzed to determine the percentage of DiI‐positive cells. To assess EGFP expression following transfection with HNP nanoparticles, B16F10 cells were harvested at 0, 24, 48, 72, and 96 h, resuspended in 100 µL of PBS, and the proportion of EGFP‐positive cells was quantified by flow cytometry.

### 5.11 Analysis of PD‐1 Expression Following HNP_PD‐1_ Transfection

CLSM and Western blot were performed to determine whether HNP_PD‐1_ nanoparticles could specifically drive PD‐1 expression in tumor cells in vitro. Briefly, B16F10 and HEK293T cells were seeded into 24‐well plates (5 × 10^4^ cells per well) and transfected with HNP_PD‐1_ nanoparticles. After 72 h, the cells were stained with Cy3‐conjugated anti‐PD‐1 antibody, and nuclei were counterstained with Hoechst 33342. The samples were then imaged by CLSM. For western blot analysis, whole‐cell lysates from HNP_PD‐1_‐transfected B16F10 and HEK293T cells were separated by 10% SDS‐PAGE and transferred onto PVDF membrane. The membrane was blocked with 5% skim milk for 1 h at room temperature and subsequently incubated overnight at 4°C with an anti‐PD‐1 primary antibody. Subsequently, the membrane was incubated with a secondary antibody at 4°C for 1 h and visualized. To comparatively assess PD‐1 and CD9 (an EVs marker) expression, the same protocol was applied to whole‐cell lysates and EVs derived from both un‐transfected and HNP_PD‐1_‐transfected B16F10 cells.

### 5.12 PD‐1^+^ EVs Bind to Tumor Cells and Relieve T‐Cell Exhaustion In Vitro

To investigate the binding of PD‐1^+^ EVs to PD‐L1 on the surface of tumor cells, B16F10 cells were inoculated into bottomless culture slides and cultured overnight. The cells were then treated with CellMask Green‐labeled PD‐1^+^ EVs; a blocking group was pre‐incubated with an anti‐PD‐1 antibody to assess binding specificity. Following EVs incubation, non‐specific binding sites were blocked with bovine serum albumin (BSA) for 1 h at room temperature. The cells were subsequently stained with Cy5‐conjugated anti‐PD‐L1 antibody for 30 min at room temperature to visualize cellular PD‐L1. Finally, nuclei were stained with Hoechst 33342, and the samples were imaged by CLSM.

To evaluate the capacity of PD‐1^+^ EVs to relieve T‐cell immunosuppression in vitro, mouse CD8^+^ T cells were isolated from splenocytes using a mouse CD8^+^ T cell selection kit (BioLegend, USA). The purified CD8^+^ T cells were first stimulated with an anti‐CD3 antibody (5 µg/mL) for 24 h. Subsequently, they were incubated for 72 h with EVs derived from B16F10 cells, PD‐1^+^ EVs, or PD‐1^+^ EVs pre‐blocked with anti‐PD‐1 antibody. Following this co‐culture period, the cell suspensions were centrifuged at 1500 g for 10 min, and the supernatants were collected. Levels of the immune factors IL‐2, TNF‐α, and IFN‐γ in the supernatant were then quantified using corresponding ELISA kits.

### 5.13 Formation and Characterization of Hydrogel@HNP

A fibrin hydrogel was prepared by mixing equal volumes of a 10 mg/mL fibrinogen solution and a 20 U/mL thrombin solution at 37°C, resulting in gelation within approximately 1 min. The morphology of hydrogel was characterized by SEM, and the rheological properties of the hydrogel were analyzed using a rheometer. To fabricate HNP‐encapsulated fibrin gel (hydrogel@HNP), HNP were first dispersed in the fibrinogen solution prior to mixing with the thrombin solution using the same protocol. And the encapsulation of DiI‐labeled HNP within the hydrogel matrix was confirmed by CLSM. For the in vitro release study, hydrogel@HNP was incubated in PBS at 37°C under different pH conditions (pH 6.5 and 7.4) to investigate HNP release behavior. To evaluate in vivo release, DiI‐labeled free HNP or hydrogel@HNP‐DiI was administered via subcutaneous injection. Fluorescence signals were subsequently monitored using IVIS spectral imaging system.

### 5.14 In Vivo Detection of Tumor Recurrence and Early Tumor Detection

All animal experiments were conducted in accordance with ethical guidelines and approved by the Life Sciences Ethical Review Committee of Zhengzhou University (Ethical Approval No. yxyllsc20240111). To establish a residual microtumor model, 1 × 10^6^ Luc‐B16F10 cells were inoculated into the right flank of each mouse. When tumor volumes reached approximately 100 mm^3^ (tumor size was calculated using the formula: width^2^ × length × 0.5), the tumor was completely resected, and 1 × 10^4^ Luci‐B16F10 cells were injected to simulate post‐surgical recurrence. Mice were then randomly divided into groups and implanted with the following formulations: hydrogel, hydrogel@HNP_miR‐26a_, or hydrogel@HNP_E‐miR‐26a_. After 7 or 8 days, the mice were imaged using an IVIS spectral imaging system. Subsequently, miRNA expression levels in both tumor tissues and plasma‐derived EVs were quantified via RT‐qPCR.

In a parallel experiment, a separate cohort of mice with residual microtumors was implanted with hydrogel, hydrogel@AAV_miR‐26a_, or hydrogel@AAV_E‐miR‐26a_. miRNA expression in plasma EVs from this cohort was analyzed using our previously developed topologically constrained DNA‐mediated one‐pot CRISPR assay. To evaluate the feasibility of early tumor detection, additional model groups were established by subcutaneous inoculating varying numbers of Luci‐B16F10 cells (5 × 10^3^, 5 × 10^4^, 5 × 10^5^, 5 × 10^6^, and 5 × 10^7^ cells).  Tumor growth was monitored using an IVIS spectral imaging system, and miRNA expression in plasma EVs was analyzed using our previously developed topologically constrained DNA‐mediated one‐pot CRISPR assay.

### 5.15 In Vivo PD‐1^+^ EVs Combination With RT Prevents Postoperative Melanoma Recurrence

To evaluate the therapeutic efficacy, C57BL/6J mice with postoperative residual microtumors were randomly divided into four groups (n = 10 per group): hydrogel (control), hydrogel + RT (6 Gy per cycle, 3 cycles), hydrogel@HNP_PD‐1_, and hydrogel@HNP_PD‐1_ + RT (6 Gy per cycle, 3 cycles). RT was initiated two days post‐surgery and administered every other day for a total of three cycles. Tumor growth was monitored via IVIS spectral imaging system, and body weight was measured regularly throughout the treatment period. Upon conclusion of the treatment on day 20, five mice from each group were euthanized. Major organs (heart, liver, spleen, lungs, and kidneys) and tumor tissues were harvested for subsequent histological and immunohistochemical analysis, including H&E staining, Ki67 proliferation assay, and fluorescent TUNEL apoptosis assay. The complete blood counts analysis was used to evaluate the biosafety of different groups. The remaining mice were maintained for survival monitoring, and their survival time was recorded.

### 5.16 Immunophenotyping of Tumor‐Infiltrating T Cells by Flow Cytometry

The obtained tumor tissues were sectioned. Approximately 0.2 g of tumor tissue was then digested in a mixture containing 10 µL of enzyme R, 100 µL of enzyme D, 12.5 µL of enzyme A, and 2.35 mL of RPMI 1640 medium. The digestion was performed at 37°C for 40 min with constant shaking. The resulting digest was filtered through a 70 µm strainer and centrifuged for 5 min to collect the pellet. The pellet was resuspended to generate a single‐cell suspension. For T‐cells immunophenotyping, cells were stained with a panel of fluorescently conjugated antibodies, including PE/Cyanine7 anti‐mouse CD45, FITC anti‐mouse CD3, APC anti‐mouse CD8a, and Brilliant Violet 421 anti‐mouse GzmB. Following two washes with PBS, the cells were resuspended in 100 µL of PBS and kept on ice prior to analysis. Flow cytometry was performed immediately thereafter, and the acquired data were analyzed using FlowJo software (version 10.4).

### 5.17 Cytokine Level Assay

To quantify cytokine levels within the tumor tissue, samples were homogenized in 9‐fold volumes of saline based on tissue weight. Homogenization was performed on ice using mechanical homogenizer to generate homogenate. The homogenate was then centrifuged at 3000 g for 10 min to collect the supernatant. Levels of TNF‐α, IL‐6, IFN‐γ, GzmB, and perforin in the supernatant were subsequently analyzed using corresponding commercial ELISA kits according to the instructions.

### 5.18 Statistical Analysis

All data were presented as mean ± standard deviation. Statistical figures were prepared using GraphPad Prism software (version 9.0). Inter‐group statistical differences were analyzed using one‐way analysis of variance (ANOVA) followed by a multiple comparisons test. Comparisons between two groups were conducted using Student's *t*‐tests. Statistical significance was defined as ^*^ *p* < 0.05, ^**^ *p* < 0.01, ^***^ *p* < 0.001, and ^****^ *p* < 0.0001. And “ns” indicated no significant difference.

## Conflicts of Interest

The authors declare no conflicts of interest.

## Supporting information




**Supporting File**: advs75136‐sup‐0001‐SuppMat.docx.

## Data Availability

The data that support the findings of this study are available in the supplementary material of this article.
