# Supplementary material for: Early Detection and Inhibition of Post‐Surgical Cancer Recurrence by Synthetic Extracellular Vesicles
Source: Adv Sci (Weinh). 2026 Apr 9;13(36):e23388. doi: 10.1002/advs.202523388 (PMC13317606; doi:10.1002/advs.202523388)
Supplement: Supplementary file 1 — Supporting File: advs75136‐sup‐0001‐SuppMat.docx. [file ADVS-13-e23388-s001.docx]

Supporting Information

Early detection and inhibition of post-surgical cancer recurrence by synthetic extracellular vesicles

Junli Zhang, Wenjia Chang, Ruolin Hu, Peiyan Su, Qin Zhou, Yanan Li*, Zhenzhong Zhang *, and Kaixiang Zhang *

J. Zhang, W. Chang, R. Hu, P. Su

School of Pharmaceutical Sciences, Zhengzhou University, Henan Key Laboratory of Nanomedicine for Targeting Diagnosis and Treatment, Zhengzhou, 450001, China;

Q. Zhou

Institute of Biomedical Engineering, College of Life Sciences, Qingdao University, Qingdao, 266071, China.

Y. Li, Z. Zhang

School of Pharmaceutical Sciences, Zhengzhou University, Henan Key Laboratory of Nanomedicine for Targeting Diagnosis and Treatment, Zhengzhou, 450001, China;

E-mail: lyn0122@zzu.edu.cn; zhangzhenzhong@zzu.edu.cn

K. Zhang

School of Pharmaceutical Sciences, Zhengzhou University, Henan Key Laboratory of Nanomedicine for Targeting Diagnosis and Treatment, State Key Laboratory of Esophageal Cancer Prevention and Treatment, Tianjian Laboratory of Advanced Biomedical Sciences, Zhengzhou, 450001, China;

Key Laboratory of Advanced Drug Preparation Technologies, Ministry of Education of China, Zhengzhou 450001, China;

E-mail: [zhangkx@zzu.edu.cn](mailto:zhangkx@zzu.edu.cn)

**List of Contents**

[Figure S1. Characterization of AAV. 3](#_Toc225436554)

[Figure S2. Characterization of control EVs, miR-26 EVs, and E-miR-26a EVs derived from AAV-transfected and un-transfected B16F10 cells. 4](#_Toc225436555)

[Figure S3. RT-qPCR analysis of the expression of miR-26a and E-miR-26a in B16F10 cells and their secreted EVs. 4](#_Toc225436556)

[Figure S4. Schematic illustration of a topologically constrained DNA-mediated one-pot CRISPR assay for ultrasensitive detection of EVs miRNA. 5](#_Toc225436557)

[Figure S5. RT-qPCR quantification of E-miR-26a level in EVs from various tumor cells lines and normal cell lines following AAV](#_Toc225436558)_[E-miR-26a](#_Toc225436558)_ [transfection 5](#_Toc225436558)

[Figure S6. Characterization of alginate hydrogel. 6](#_Toc225436559)

[Figure S7. The distribution of AAV/Cy5 within alginate hydrogel and release profiles. 6](#_Toc225436560)

[Figure S8. Bioluminescence imaging of mice inoculated Luci](#_Toc225436561)^[+](#_Toc225436561)^ [B16F10](#_Toc225436561)[cells. 7](#_Toc225436561)

[Figure S9.](#_Toc225436562) *[In vivo](#_Toc225436562)* [bioluminescence imaging of tumor growth in mice inoculated with different numbers of Luci](#_Toc225436562)^[+](#_Toc225436562)^ [B16F10 cells. 7](#_Toc225436562)

[Figure S10. Characterization of HNP. 8](#_Toc225436563)

[Figure S11. The cellular uptake of HNP](#_Toc225436564)_[E-miR-26a](#_Toc225436564)_ [and EGFP expression in B16F10 cells. 8](#_Toc225436564)

[Figure S12. Characterization of control EVs, miR-26 EVs, and E-miR-26a EVs derived from HNP-transfected and un-transfected B16F10 cells. 9](#_Toc225436565)

[Figure S13. Characterization of fibrin hydrogel 10](#_Toc225436566)

[Figure S14.](#_Toc225436567) *[In vivo](#_Toc225436567)* [tumor recurrence detection. 11](#_Toc225436567)

[Figure S15. H&E staining, Ki67 immunostaining and TUNEL staining of tumor tissue 12](#_Toc225436568)

[Figure S16. H&E staining of major organs and complete blood counts analysis 13](#_Toc225436569)

[Figure S17. Representative flow cytometric analysis of CD3](#_Toc225436570)^[+](#_Toc225436570)^ [CD45](#_Toc225436570)^[+](#_Toc225436570)^ [T cells, CD3](#_Toc225436570)^[+](#_Toc225436570)^ [CD4](#_Toc225436570)^[+](#_Toc225436570)^ [T cells, and CD3](#_Toc225436570)^[+](#_Toc225436570)^ [CD8](#_Toc225436570)^[+](#_Toc225436570)^ [T cells. 14](#_Toc225436570)

[Figure S18. Flow cytometric analysis images and relative quantification of GzmB⁺CD8⁺ T cells within tumors after various treatments. 15](#_Toc225436571)

[Figure S19. Plasmid maps of miR-26a, E-miR-26a and PD-1. 15](#_Toc225436572)

[Table S1. DNA sequences of Survivin promoter, TERT promoter, miR-26a, E-miR-26a, and PD-1. 16](#_Toc225436573)

[Table S2. Comparison of methods for detecting tumor recurrence. 17](#_Toc225436574)

**Results**


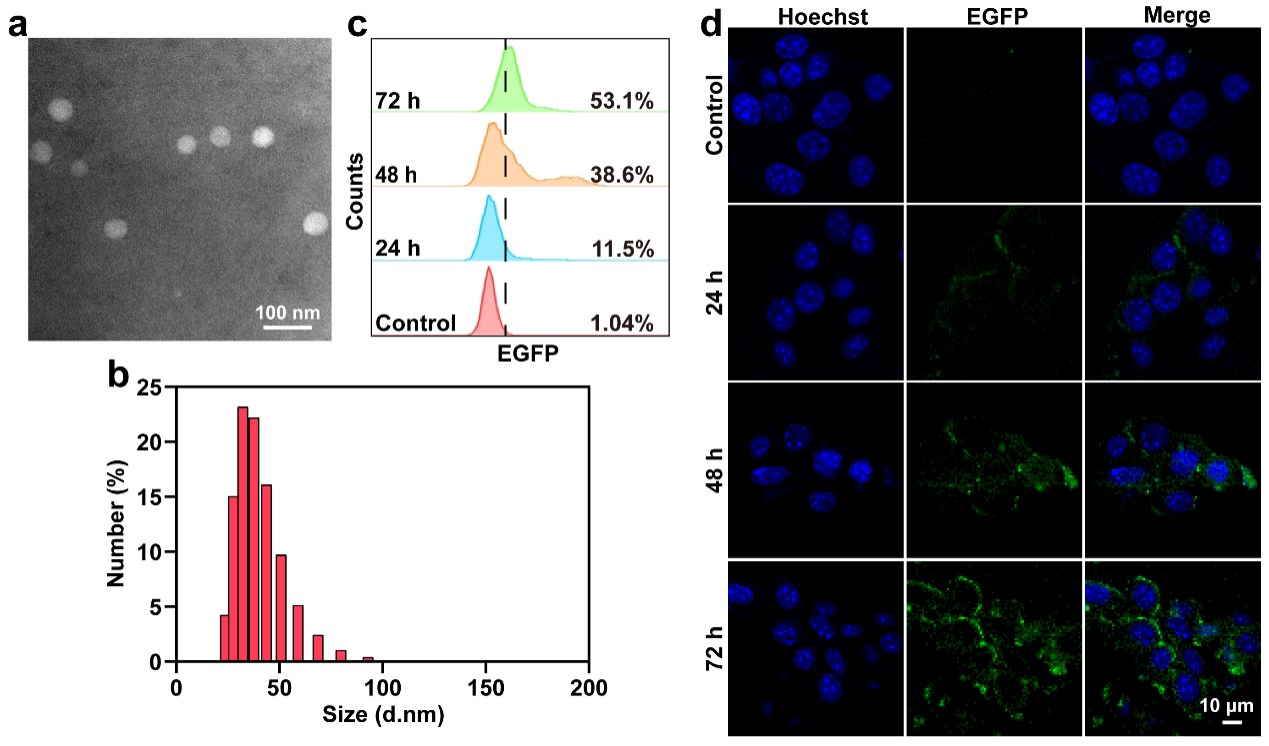


Figure S1. Characterization of AAV. (a) Representative TEM image of AAV_E-miR-26a_ vector. (b) DLS analysis of size distribution of AAV_E-miR-26a_. (c) Flow cytometry analysis and (d) fluorescence images showing the expression of EGFP in B16F10 cells after incubation with AAV_E-miR-26a_ at 0, 24, 48, and 72 h (scale bar = 10 µm).


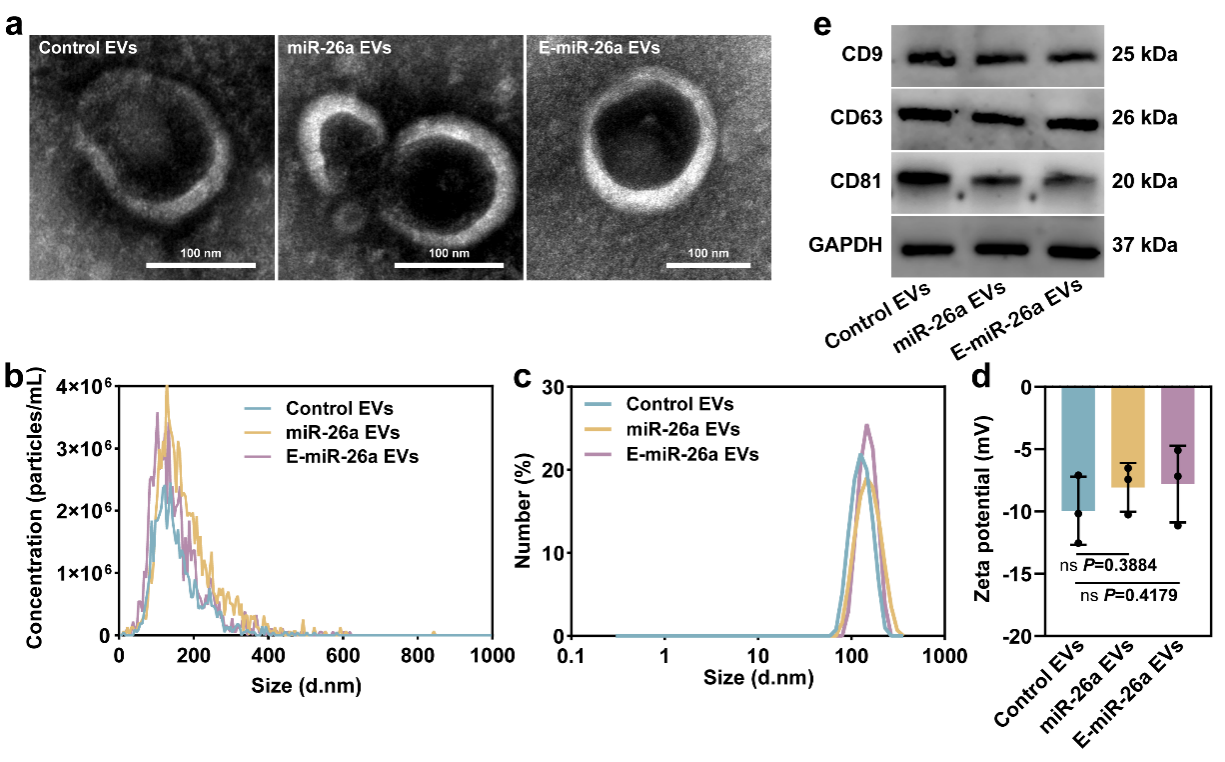


Figure S2. Characterization of control EVs, miR-26 EVs, and E-miR-26a EVs derived from AAV-transfected and un-transfected B16F10 cells. (a) Representative TEM images of control EVs, miR-26 EVs, and E-miR-26a EVs. (b) NTA measurement of size and concentration of control EVs, miR-26 EVs, and E-miR-26a EVs. (c-d) DLS analysis of (c) size distribution and (d) zeta potential of control EVs, miR-26 EVs, and E-miR-26a EVs. (e) Western blot analysis of the expression of CD9, CD63, and CD81 in control EVs, miR-26 EVs, and E-miR-26a EVs, and GAPDH was used as an internal control.


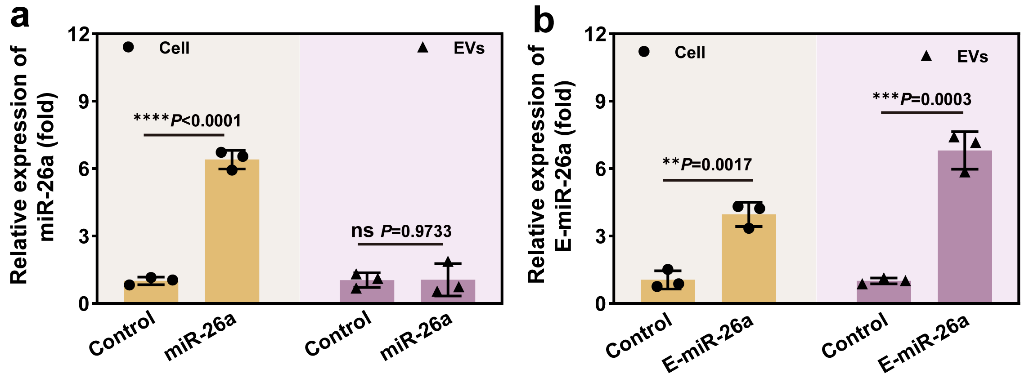


Figure S3. RT-qPCR analysis of the expression of (a) miR-26a and (b) E-miR-26a in B16F10 cells and their secreted EVs.


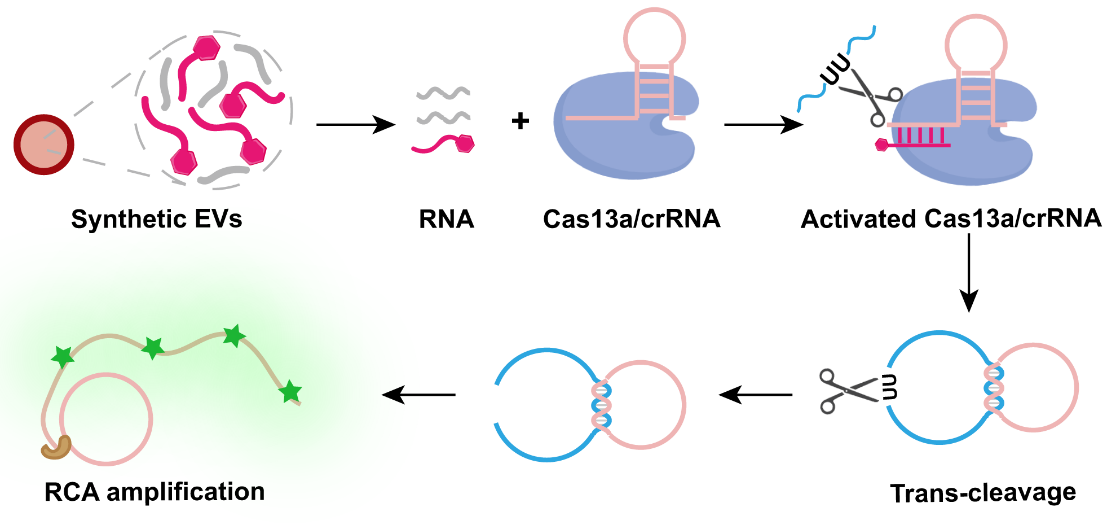


Figure S4. Schematic illustration of a topologically constrained DNA-mediated one-pot CRISPR assay for ultrasensitive detection of EVs miRNA. The CRISPR/Cas13a/crRNA system recognizes the target E-miR-26a. Upon target binding, the activated Cas13a complex exhibits trans-cleavage activity, efficiently cleaving a topologically constrained DNA ring to initiate rolling circle amplification (RCA). This cascade enables highly sensitive detection of EVs**-**derived miRNA via fluorescence signal measurement.


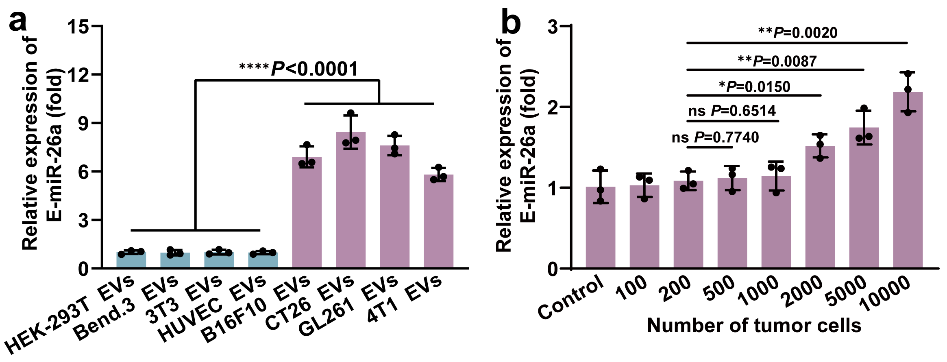


Figure S5. (a) RT-qPCR quantification of E-miR-26a level in EVs from various tumor cells lines (B16F10, CT26, GL261, 4T1 cells) and normal cell lines (HEK-293T, Bend.3, 3T3, HUVEC cells) following AAV_E-miR-26a_ transfection. (b) RT-qPCR quantification of E-miR-26a level in the EVs derived from different numbers of B16F10 cells.


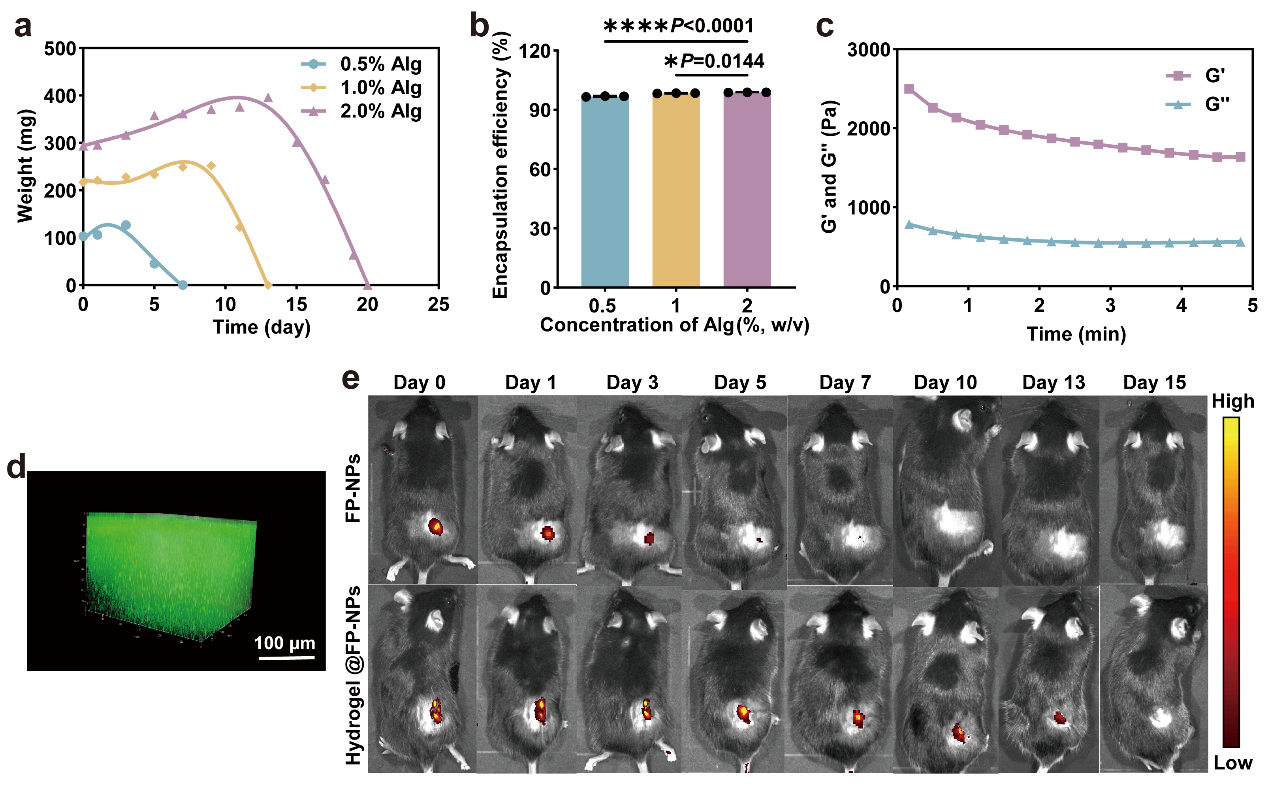


Figure S6. Characterization of alginate hydrogel. (a) Quantitative analysis of the remaining weights of alginate hydrogels prepared at different alginate concentrations. (b) Encapsulation efficiency of FP-NPs in alginate hydrogels prepared at different alginate concentrations. (c) Time-sweep curves of alginate hydrogels containing 2% alginate. (d) Fluorescence images showing the distribution of FP-NPs within alginate hydrogel containing 2% alginate. (e) *In vivo* IVIS spectral imaging of the degradation of FP-NPs and hydrogel@FP-NPs.


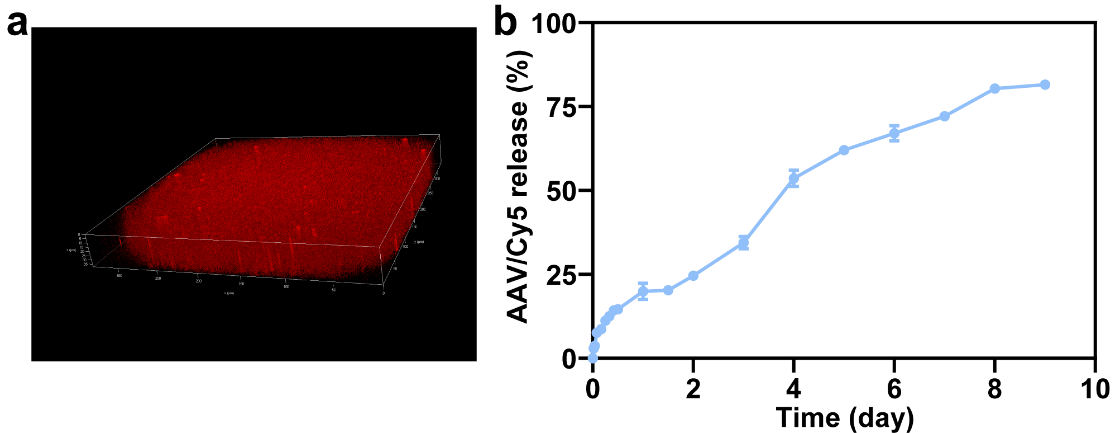


Figure S7. The distribution of AAV/Cy5 within alginate hydrogel and release profiles. (a) Fluorescence images showing the distribution of AAV/Cy5 within alginate hydrogel. (b) Time-dependent release profiles of AAV/Cy5 from sodium alginate hydrogel.


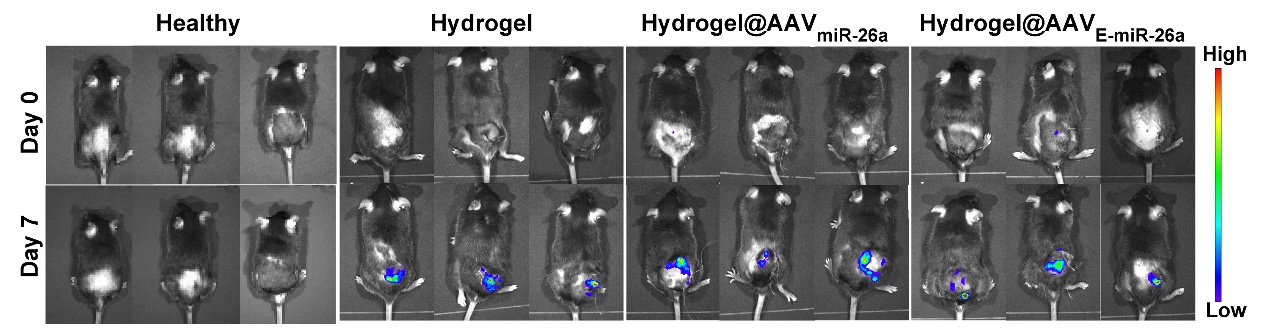


Figure S8. Bioluminescence imaging of mice inoculated Luci^+^ B16F10 cells. The primary tumor was resected and imaged on day 0. Subsequently, hydrogel, hydrogel@AAV_miR-26a_, or hydrogel@AAV_E-miR-26a_ was implanted at the surgical site, and bioluminescence imaging was repeated on day 7.


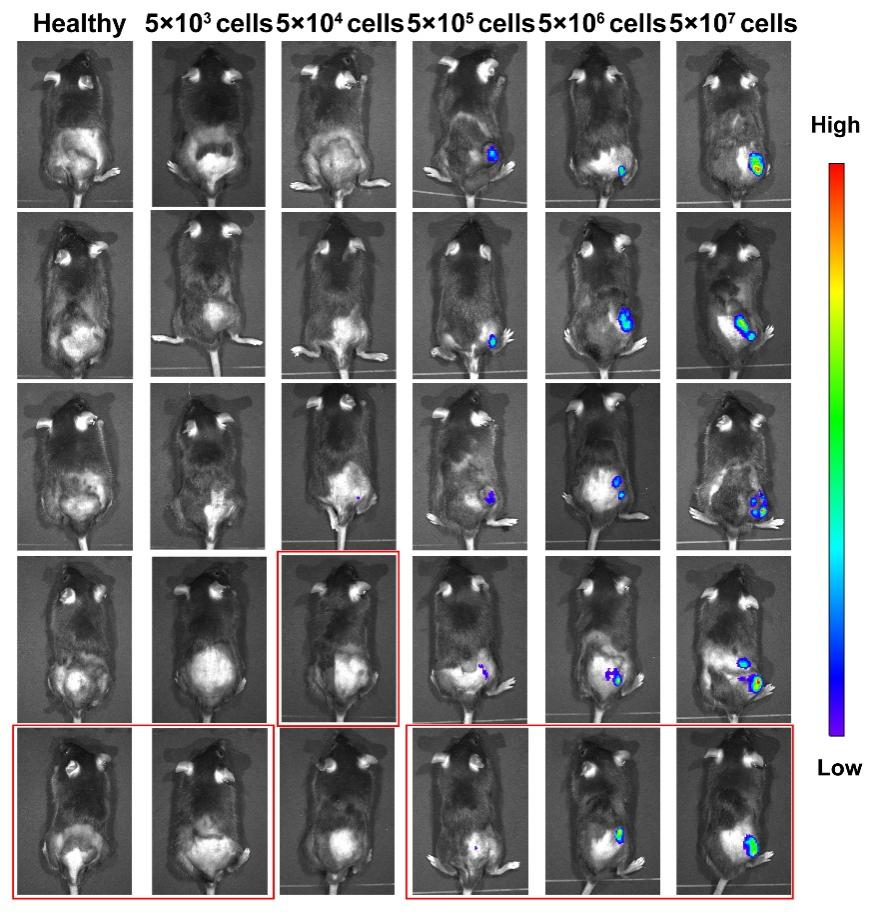


Figure S9. *In vivo* bioluminescence imaging of tumor growth in mice inoculated with different numbers of Luci^+^ B16F10 cells. The representative mice in the red box were also shown in Figure 2l.


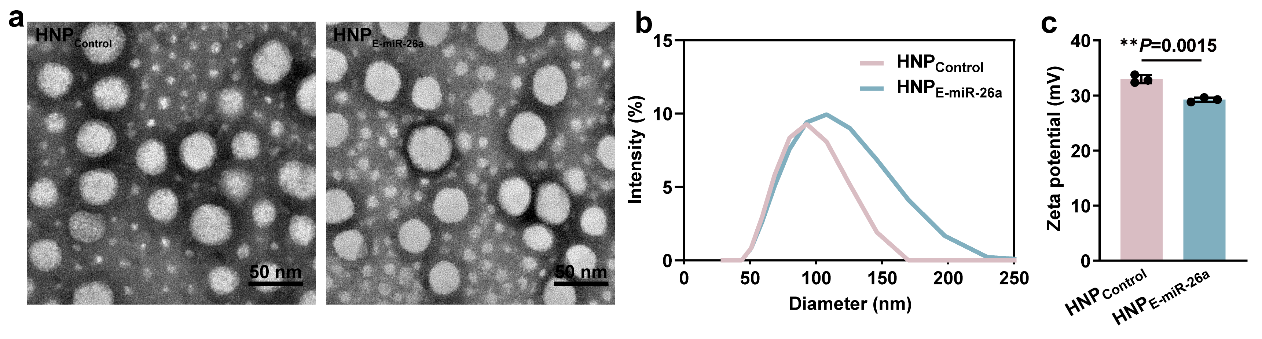


Figure S10. Characterization of HNP. (a) Representative TEM images, (b) particles size, and (c) zeta potential of blank HNP (HNP _Control_) and HNP loaded with plasmid (HNP_E-miR-26a_).


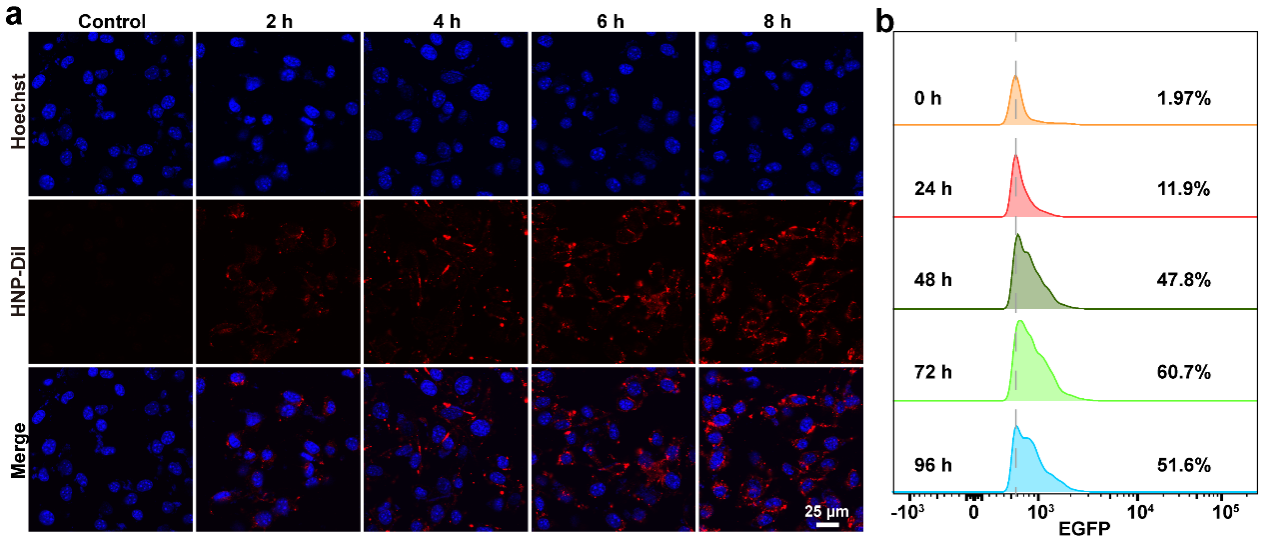


Figure S11. The cellular uptake of HNP_E-miR-26a_ and EGFP expression in B16F10 cells. (a) Time-dependent fluorescence images of DiI-labeled HNP_E-miR-26a_ uptake in B16F10 cells after co-incubation for 0, 2, 4, 6, and 8 h. (b) Flow cytometry results showing the expression of EGFP in B16F10 cells after transfection with HNP_E-miR-26a_ at 0, 24, 48, 72, and 96 h.


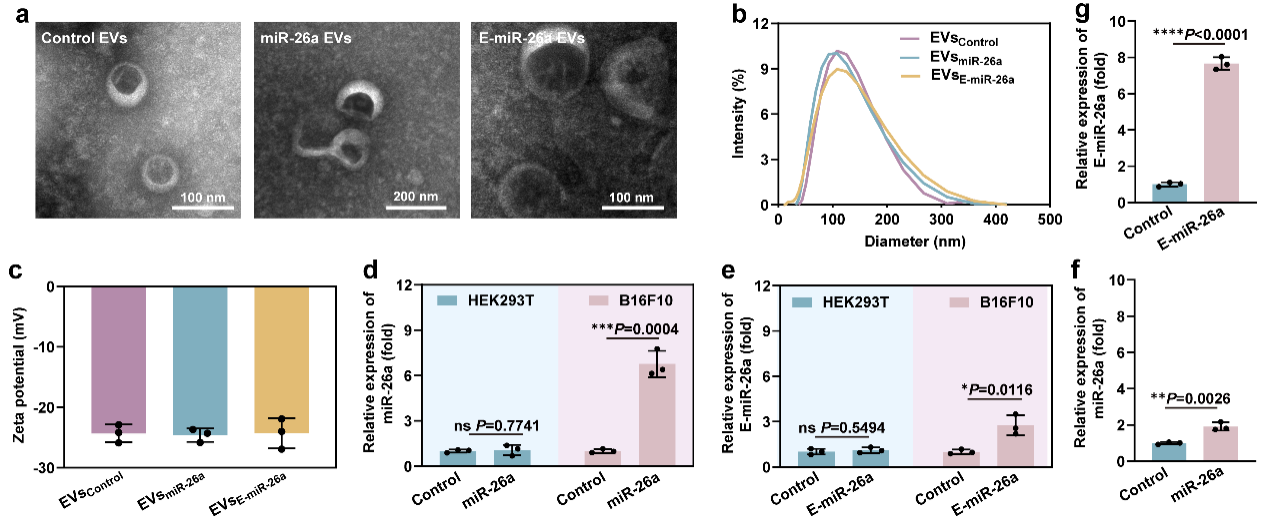


Figure S12. Characterization of control EVs, miR-26 EVs, and E-miR-26a EVs derived from HNP-transfected and un-transfected B16F10 cells. (a) Representative TEM images of control EVs, miR-26 EVs, and E-miR-26a EVs. (b-c) DLS analysis of (b) size distribution and (c) zeta potential of control EVs, miR-26 EVs, and E-miR-26a EVs. (d-e) Relative expression of (d) miR-26a or (e)E-miR-26a in EVs derived from B16F10 cells and HEK293T cells after transfection with HNP_miR-26a_ or HNP_E-miR-26a_. (f-g) The enrichment of (f) miR-26a or (g) E-miR-26a in B16F10 melanoma cells derived EVs.


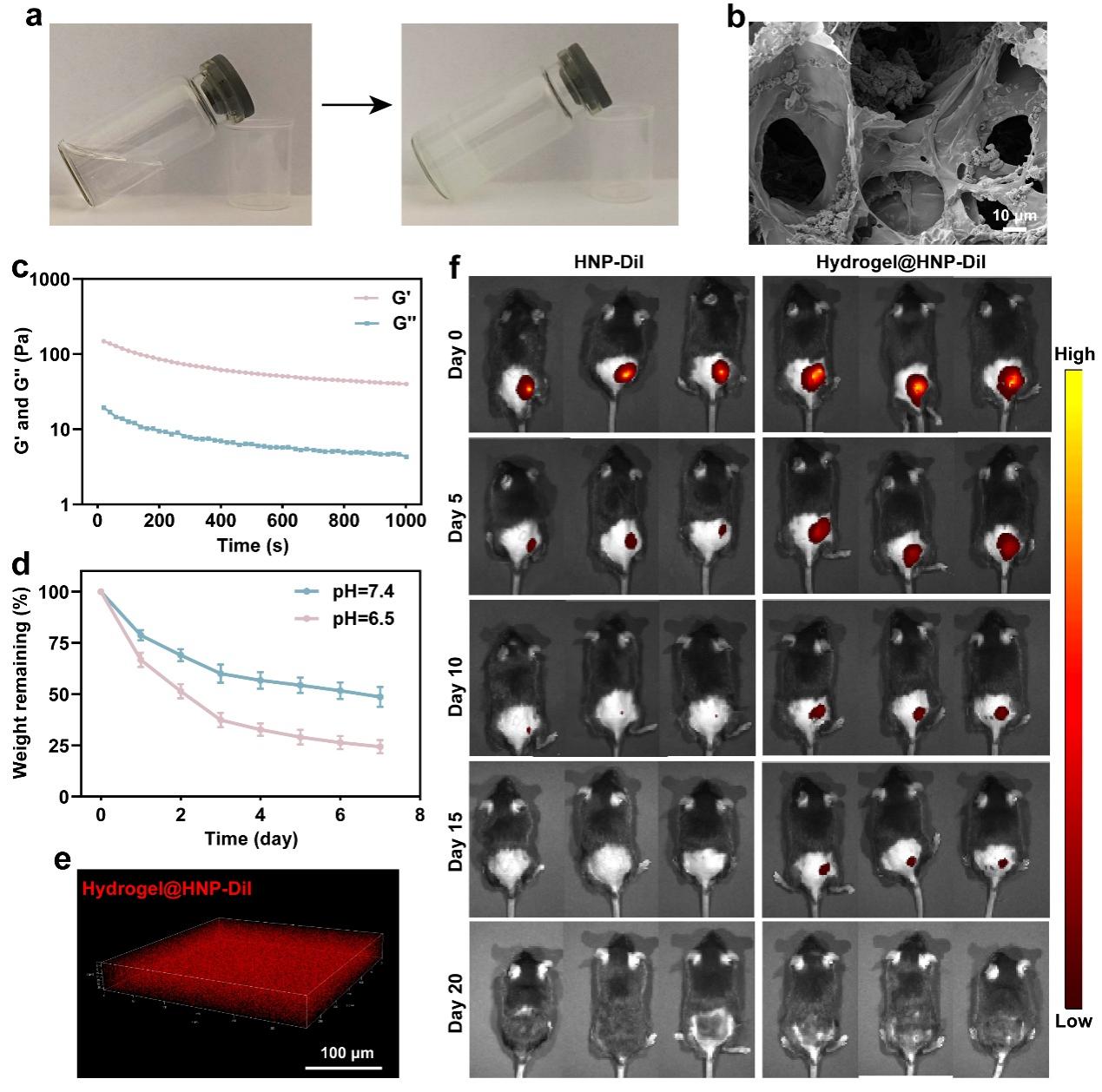


Figure S13. Characterization of fibrin hydrogel. (a) Photographs of solid fibrin hydrogel formation after mixing fibrinogen with thrombin. (b) SEM image of fibrin hydrogel after freeze-drying. (c) Time sweep measurement of G’ and G’’ during the mixing of fibrinogen and thrombin showing an ultrafast sol-gel transition. (d) Quantitative analysis of remaining weight of fibrin hydrogel at pH 7.4 or pH 6.5. (e) Fluorescence images showing the distribution of HNP-DiI within fibrin hydrogel. (f) *In vivo* IVIS spectral imaging of the degradation of HNP-DiI and hydrogel@HNP-DiI at the indicated time points.


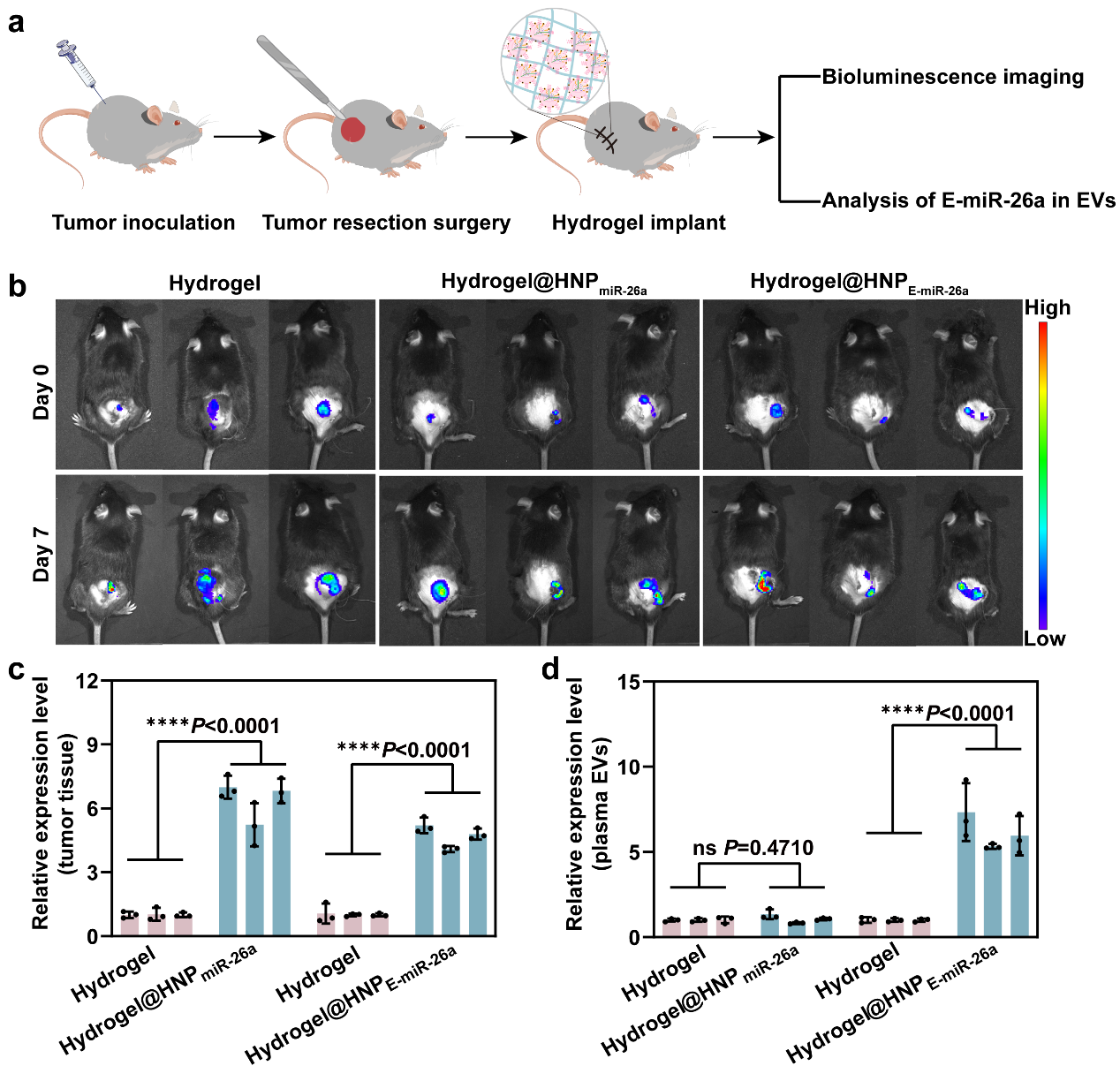


Figure S14. *In vivo* tumor recurrence detection. (a) Schematic of tumor recurrence detection. Tumor recurrence was detected via bioluminescence imaging and quantification of E-miR-26a levels in plasma EVs. (b) *In vivo* bioluminescence images of tumors in mice inoculated with Luci^+^ B16F10 cells. Following primary tumor resection (Day 0), hydrogel, hydrogel@HNP_miR-26a_, or hydrogel@HNP_E-miR-26a_ was implanted at the surgical site, and bioluminescence imaging was repeated on day 7. (c-d) Relative expression of miR-26a or E-miR-26a in (c) tumor tissues and (d) plasma-derived EVs.


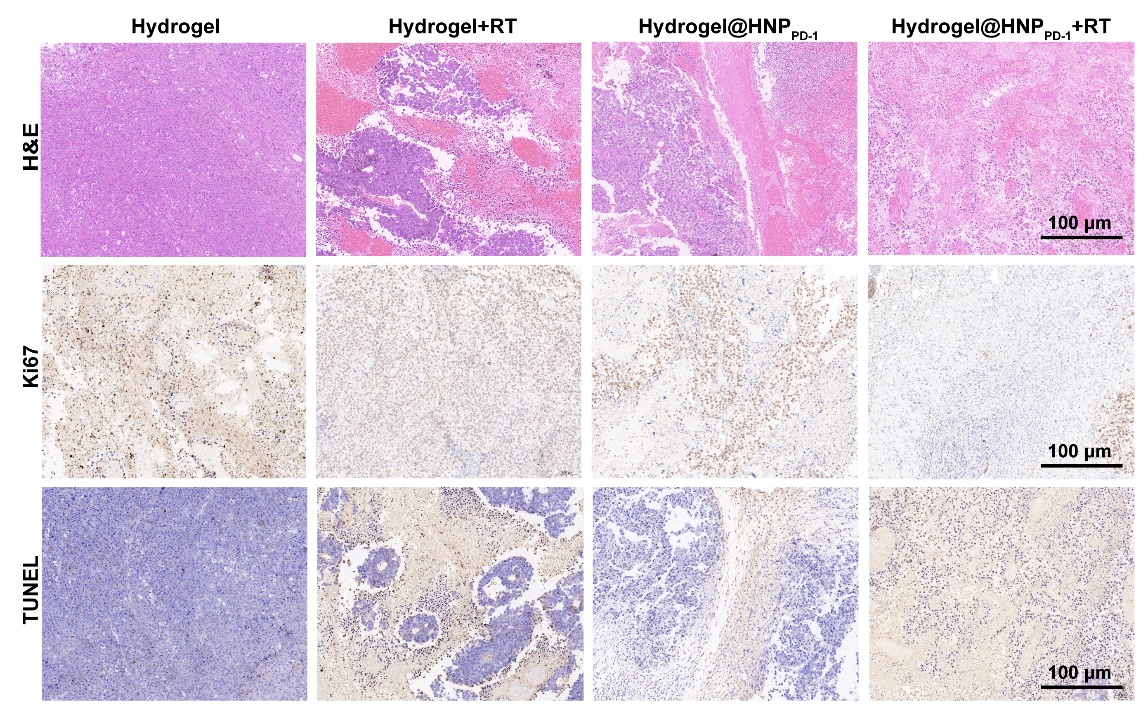


Figure S15. Representative images of tumor tissue sections after different treatments, showing H&E staining, Ki67 immunostaining, and TUNEL staining.


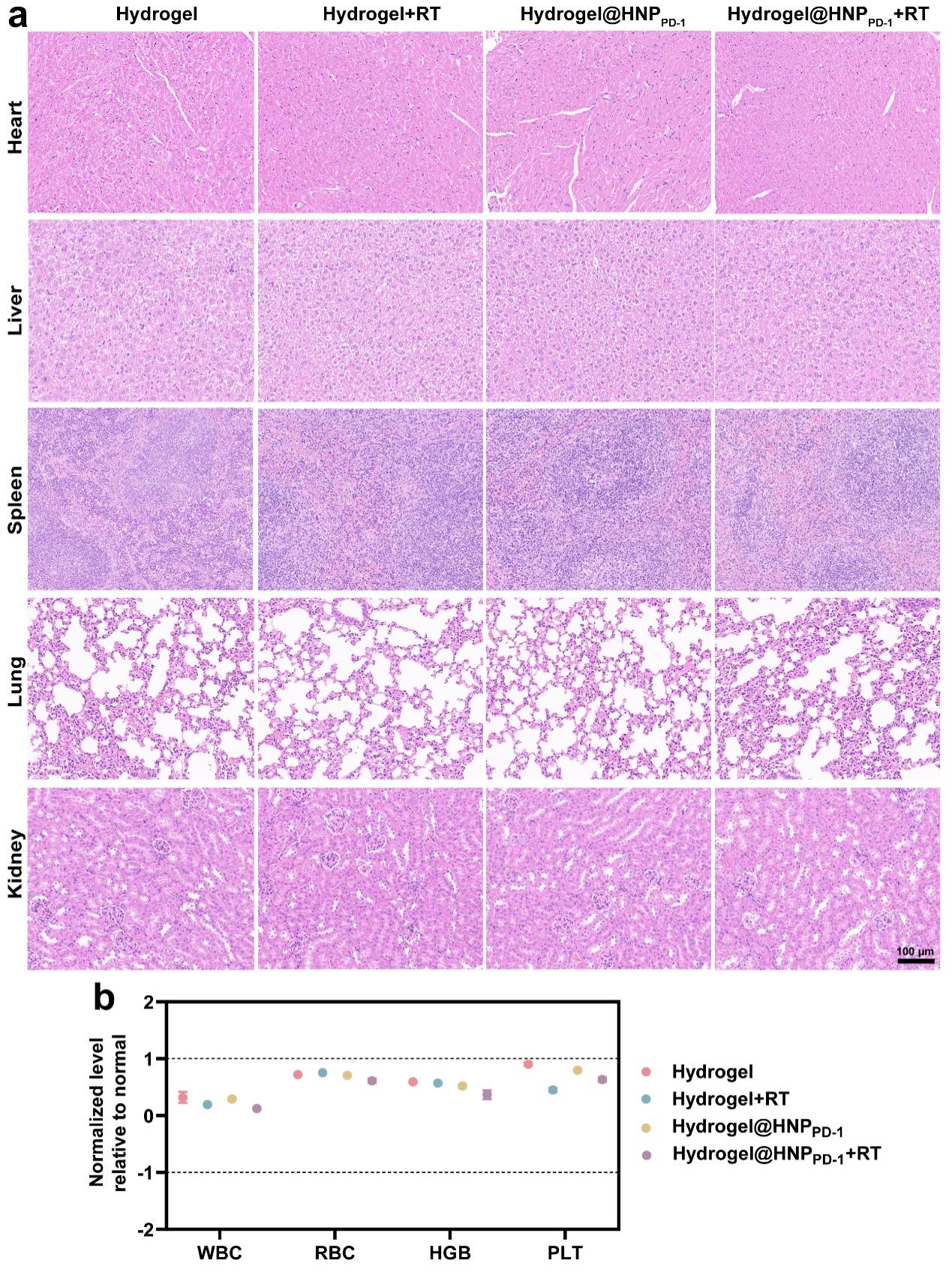


Figure S16. (a) Representative H&E staining of major organs after treated with hydrogel, hydrogel + RT, hydrogel@HNP_PD-1_, and hydrogel@HNP_PD-1_ +RT. (b) Complete blood counts analysis after different treatments.


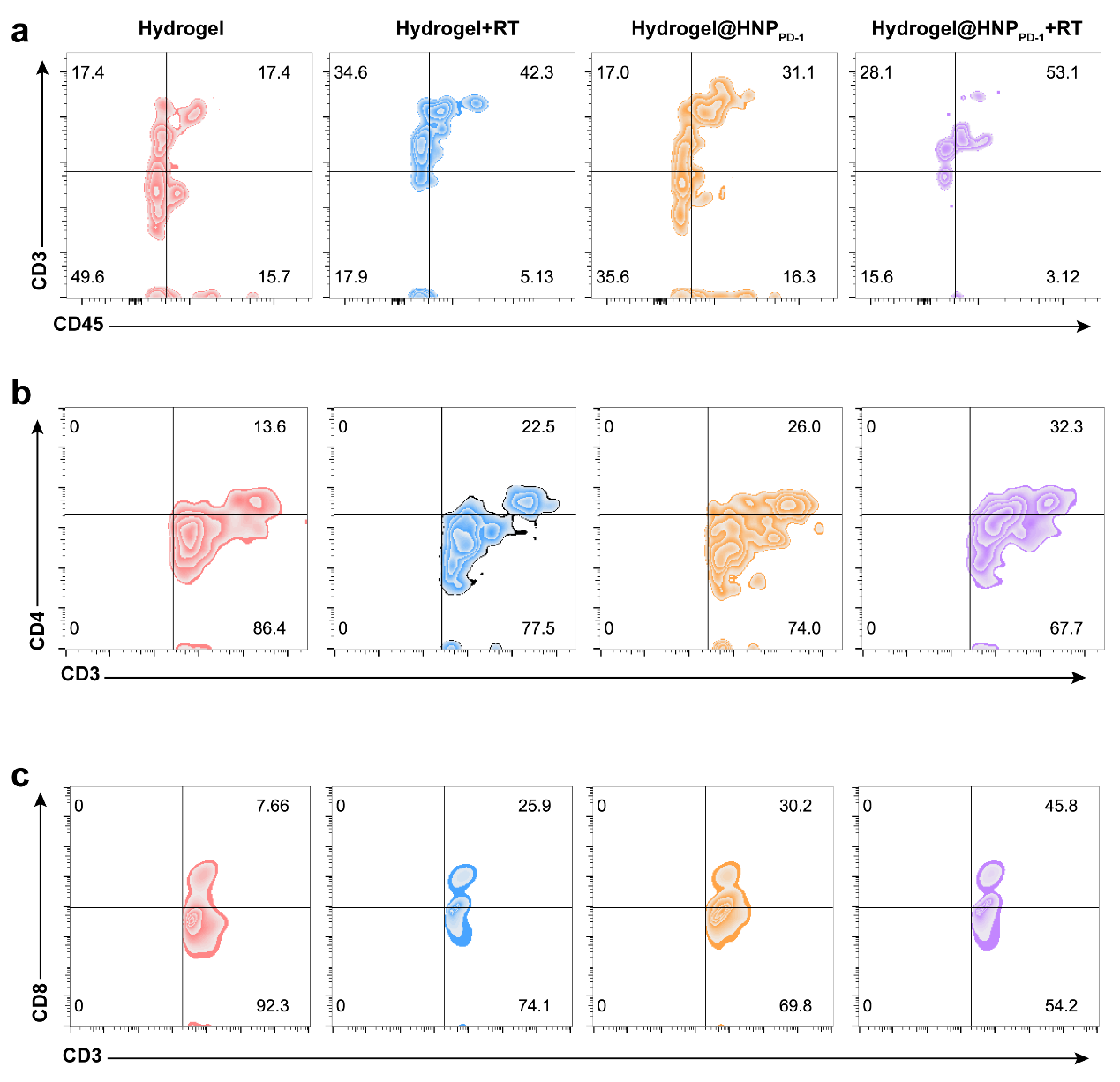


Figure S17. Representative flow cytometric analysis of (a) CD3^+^ CD45^+^ T cells, (b) CD3^+^ CD4^+^ T cells, and (c) CD3^+^ CD8^+^ T cells.


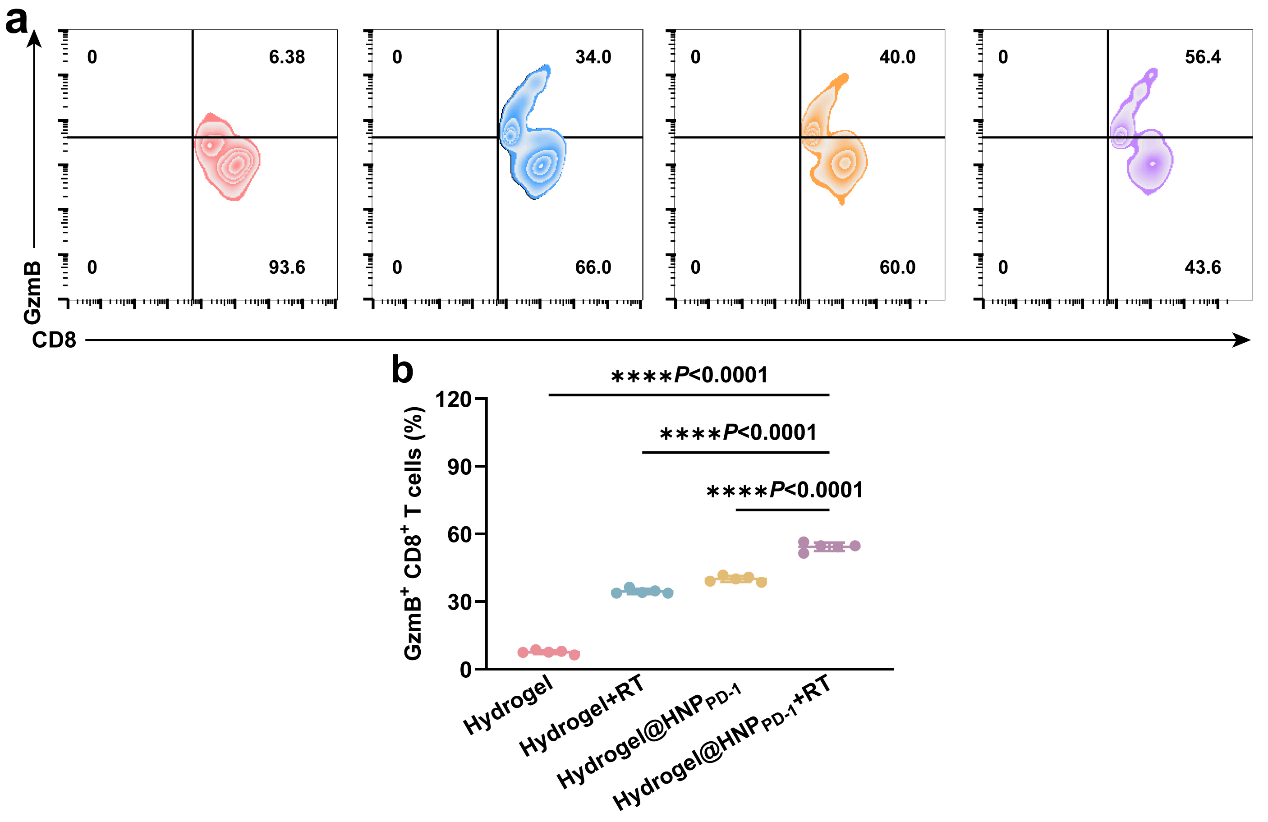


Figure S18. (a) Flow cytometric analysis images and (b) relative quantification of GzmB⁺CD8⁺ T cells within tumors after various treatments.


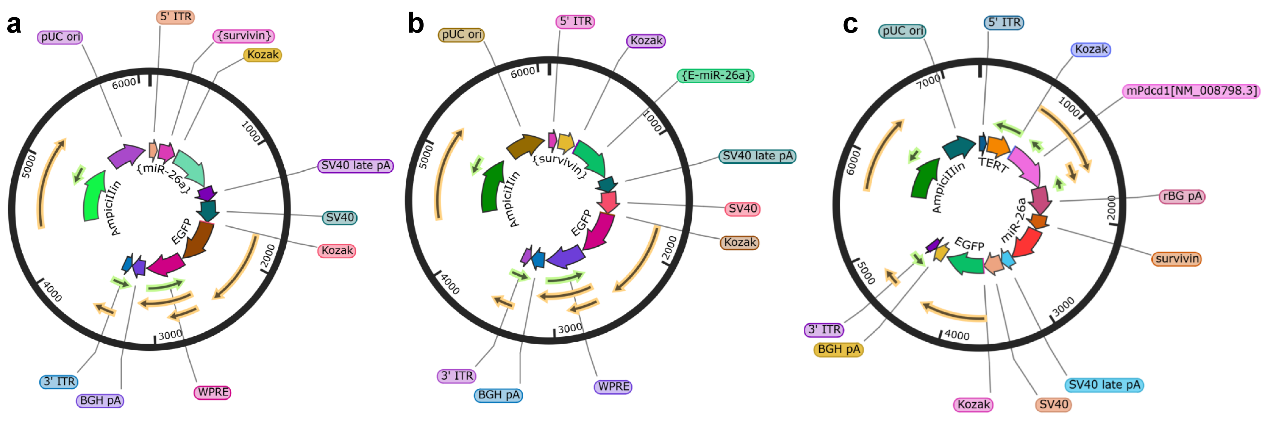


Figure S19. Plasmid maps of (a) miR-26a, (b) E-miR-26a and (c) PD-1 (encoded by the Pdcd1 gene).

Table S1. DNA sequences of Survivin promoter, TERT promoter, miR-26a, E-miR-26a, and PD-1.

| Name | Sequence（5′-3′） |
| --- | --- |
| TERT promoter  (455 bps) | TGGCCCCTCCCTCGGGTTACCCCACAGCCTAGGCCGATTCGACCTCTCTCCGCTGGGGCCCTCGCTGGCGTCCCTGCACCCTGGGAGCGCGAGCGGCGCGCGGGCGGGGAAGCGCGGCCCAGACCCCCGGGTCCGCCCGGAGCAGCTGCGCTGTCGGGGCCAGGCCGGGCTCCCAGTGGATTCGCGGGCACAGACGCCCAGGACCGCGCTTCCCACGTGGCGGAGGGACTGGGGACCCGGGCACCCGTCCTGCCCCTTCACCTTCCAGCTCCGCCTCCTCCGCGCGGACCCCGCCCCGTCCCGACCCCTCCCGGGTCCCCGGCCCAGCCCCCTCCGGGCCCTCCCAGCCCCTCCCCTTCCTTTCCGCGGCCCCGCCCTCTCCTCGCGGCGCGAGTTTCAGGCAGCGCTGCGTCCTGCTGCGCACGTGGGAAGCCCTGGCCCCGGCCACCCCCGCG |
| Pdcd1/ PD-1  (867 bps) | ATGTGGGTCCGGCAGGTACCCTGGTCATTCACTTGGGCTGTGCTGCAGTTGAGCTGGCAATCAGGGTGGCTTCTAGAGGTCCCCAATGGGCCCTGGAGGTCCCTCACCTTCTACCCAGCCTGGCTCACAGTGTCAGAGGGAGCAAATGCCACCTTCACCTGCAGCTTGTCCAACTGGTCGGAGGATCTTATGCTGAACTGGAACCGCCTGAGTCCCAGCAACCAGACTGAAAAACAGGCCGCCTTCTGTAATGGTTTGAGCCAACCCGTCCAGGATGCCCGCTTCCAGATCATACAGCTGCCCAACAGGCATGACTTCCACATGAACATCCTTGACACACGGCGCAATGACAGTGGCATCTACCTCTGTGGGGCCATCTCCCTGCACCCCAAGGCAAAAATCGAGGAGAGCCCTGGAGCAGAGCTCGTGGTAACAGAGAGAATCCTGGAGACCTCAACAAGATATCCCAGCCCCTCGCCCAAACCAGAAGGCCGGTTTCAAGGCATGGTCATTGGTATCATGAGTGCCCTAGTGGGTATCCCTGTATTGCTGCTGCTGGCCTGGGCCCTAGCTGTCTTCTGCTCAACAAGTATGTCAGAGGCCAGAGGAGCTGGAAGCAAGGACGACACTCTGAAGGAGGAGCCTTCAGCAGCACCTGTCCCTAGTGTGGCCTATGAGGAGCTGGACTTCCAGGGACGAGAGAAGACACCAGAGCTCCCTACCGCCTGTGTGCACACAGAATATGCCACCATTGTCTTCACTGAAGGGCTGGGTGCCTCGGCCATGGGACGTAGGGGCTCAGCTGATGGCCTGCAGGGTCCTCGGCCTCCAAGACATGAGGATGGACATTGTTCTTGGCCTCTTTGA |
| Survivin promoter (268 bps) | GTTCTTTGAAAGCAGTCGAGGGGGCGCTAGGTGTGGGCAGGGACGAGCTGGCGCGGCGTCGCTGGGTGCACCGCGACCACGGGCAGAGCCACGCGGCGGGAGGACTACAACTCCCGGCACACCCCGCGCCGCCCCGCCTCTACTCCCAGAAGGCCGCGGGGGGTGGACCGCCTAAGAGGGCGTGCGCTCCCGACATGCCCCGCGGCGCGCCATTAACCGCCAGATTTGAATCGCGGGACCCGTTGGCAGAGGTGGCGGCGGCGGCATC |
| E-miR-26a (690 bps) | AAGGCCGUGGCCUCGUUCAAGUAAUC**CGGGAG**AGGCUGUGCAGGUCCCAAGGGGCCUCUUCUUGGUUACUUGCACGGGGACGCGGGCCUG |
| miR-26a (690 bps) | AAGGCCGUGGCCUCGUUCAAGUAAUCCAGGAUAGGCUGUGCAGGUCCCAAGGGGCCUAUUCUUGGUUACUUGCACGGGGACGCGGGCCUG |

Table S2. Comparison of methods for detecting tumor recurrence.

| Method | Detection limit | Advantages | Disadvantages | References |
| --- | --- | --- | --- | --- |
| MRI | Minimum diameter ≥ 5 mm | 91.7% sensitivity and 87% specificity | Restrict in distinguishing postoperative inflammation, radiation necrosis from tumor recurrence | [1-3] |
| CT | Spatial resolution of 0.125 mm (125 µm) / 40 lp/cm | 75% sensitivity and 87% specificity | X-rays possess radiation | [4, 5] |
| ctDNA | ~0.6 copies/mL plasma | 100% sensitivity (13/13) and 98% specificity (48/49) | Using plasma multiplex polymerase chain reaction next-generation sequencing, requirement of laboratory equipment and professional technicians | [6] |
| CTC | at least 1 CTC in 3 ml blood samples | CTC presence at baseline independently predicts recurrence (OR = 8.40, p < 0.0001) | The operational complexity of CTC isolation and identification | [7] |
| Synthetic biomarkers EVs | ~5×10⁵ tumor cells | High sensitivity and early detection capability | Detection depends on the transfection of tumor cells, may lead to false negatives | This work |

**References**

1. Z. S. Mayo, A. Halima, J. R. Broughman, et al., "Radiation necrosis or tumor progression? A review of the radiographic modalities used in the diagnosis of cerebral radiation necrosis", Journal of Neuro-Oncology 161, no. 1, (2023): 23-31

2. S. Mulé, E. Reizine, P. Blanc-Durand, et al., "Whole-Body Functional MRI and PET/MRI in Multiple Myeloma", Cancers 12, no. 11, (2020): 3155

3. E.-H. Song, S.-Y. Lee, S. Lee, et al., "Diagnosis of Local Recurrence of Malignant Soft Tissue Tumors after Reconstructive Surgery on MRI", Journal of Clinical Medicine 12, no. 13, (2023): 4369

4. T. Flohr, and B. Schmidt, "Technical Basics and Clinical Benefits of Photon-Counting CT", Investigative Radiology 58, no. 7, (2023): 441-450

5. Y. Sasaki, Y. Kondo, T. Aoki, et al., "Use of deep learning to predict postoperative recurrence of lung adenocarcinoma from preoperative CT", International Journal of Computer Assisted Radiology and Surgery 17, no. 9, (2022): 1651-1661

6. E. Christensen, K. Birkenkamp-Demtroder, H. Sethi, et al., "Early Detection of Metastatic Relapse and Monitoring of Therapeutic Efficacy by Ultra-Deep Sequencing of Plasma Cell-Free DNA in Patients With Urothelial Bladder Carcinoma", J. Clin. Oncol. 37, no. 18, (2019): 1547-1557

7. X. Zhang, C. E. Weeramange, B. G. M. Hughes, et al., "Circulating tumour cells predict recurrences and survival in head and neck squamous cell carcinoma patients", Cellular and Molecular Life Sciences 81, no. 1, (2024): 233
